# Enforcement of entailment constraints in distributed service-based business processes^☆^

**DOI:** 10.1016/j.infsof.2013.05.001

**Published:** 2013-11

**Authors:** Waldemar Hummer, Patrick Gaubatz, Mark Strembeck, Uwe Zdun, Schahram Dustdar

**Affiliations:** a Distributed Systems Group, Vienna University of Technology, Austria; b Software Architecture Group, Faculty of Computer Science, University of Vienna, Austria; c Institute of Information Systems, New Media Lab, Vienna University of Economics and Business, Austria

**Keywords:** Identity and access management, Business process management, Entailment constraints, Service-Oriented Architecture (SOA), WS-BPEL

## Abstract

**Context:**

A distributed business process is executed in a distributed computing environment. The service-oriented architecture (SOA) paradigm is a popular option for the integration of software services and execution of distributed business processes. Entailment constraints, such as mutual exclusion and binding constraints, are important means to control process execution. Mutually exclusive tasks result from the division of powerful rights and responsibilities to prevent fraud and abuse. In contrast, binding constraints define that a subject who performed one task must also perform the corresponding bound task(s).

**Objective:**

We aim to provide a model-driven approach for the specification and enforcement of task-based entailment constraints in distributed service-based business processes.

**Method:**

Based on a generic metamodel, we define a domain-specific language (DSL) that maps the different modeling-level artifacts to the implementation-level. The DSL integrates elements from role-based access control (RBAC) with the tasks that are performed in a business process. Process definitions are annotated using the DSL, and our software platform uses automated model transformations to produce executable WS-BPEL specifications which enforce the entailment constraints. We evaluate the impact of constraint enforcement on runtime performance for five selected service-based processes from existing literature.

**Results:**

Our evaluation demonstrates that the approach correctly enforces task-based entailment constraints at runtime. The performance experiments illustrate that the runtime enforcement operates with an overhead that scales well up to the order of several ten thousand logged invocations. Using our DSL annotations, the user-defined process definition remains declarative and clean of security enforcement code.

**Conclusion:**

Our approach decouples the concerns of (non-technical) domain experts from technical details of entailment constraint enforcement. The developed framework integrates seamlessly with WS-BPEL and the Web services technology stack. Our prototype implementation shows the feasibility of the approach, and the evaluation points to future work and further performance optimizations.

## 1 Introduction

The Service-Oriented Architecture (SOA) metaphor has been elaborated by different communities to address different problem areas (such as enterprise application integration or business process management, see, e.g., [1]). Amongst others, it can be seen as a set of technology independent concepts for distributed computing environments. In this context, it has emerged as a popular paradigm for developing loosely coupled distributed systems [2], [3]. Today, Web services [4] are a commonly used technology which serves as a foundation of SOAs, as well as distributed business processes. A distributed business process is an intra-organizational or cross-organizational business process executed in a distributed computing environment (such as SOA). Business processes often require the definition and enforcement of process-related security policies. For example, such requirements result from internal business rules of an organization, or service-level agreements (SLAs) [5] with customers. In addition, numerous regulations and IT standards exist that pose compliance requirements for the corresponding systems. In particular, IT systems must comply with laws and regulations such as the Basel II/III Accords, the International Financial Reporting Standards (IFRSs), or the Sarbanes–Oxley Act (SOX). For instance, one important part of SOX compliance is to provide adequate support for definition and enforcement of process-related security policies (see, e.g., [6], [7], [8]).

Role-based access control (RBAC) [9], [10] is a de facto standard for access control in both research and industry. In the context of RBAC, roles are used to model different job positions and scopes of duty within an information system. These roles are equipped with the permissions to perform their respective tasks. Human users and other active entities (subjects) are assigned to roles according to their work profile [11], [12]. A process-related RBAC model (see, e.g., [13], [14]) enables the definition of permissions and entailment constraints for the tasks that are included in business processes. A *task-based entailment constraint* places some restriction on the subjects who can perform a task *x* given that a certain subject has performed another task *y*. Entailment constraints are an important means to assist the specification and enforcement of compliant business processes (see, e.g., [15], [16], [17], [18], [19], [20]).

Mutual exclusion and binding constraints are typical examples of entailment constraints. Mutual exclusion constraints can be subdivided in *Static Mutual Exclusion* (SME) and *Dynamic Mutual Exclusion* (DME) constraints. A SME constraint defines that two tasks (e.g. Order Supplies and Approve Payment) must never be assigned to the same role and must never be performed by the same subject (to prevent fraud and abuse). This constraint is global with respect to *all process instances* in an information system. In contrast, DME refers to individual process instances and can be enforced by defining that two tasks must never be performed by the same subject in the *same process instance*.

In contrast to mutual exclusion constraints, binding constraints define that two bound tasks must be performed by the *same* entity. In particular, a *subject-binding* constraint defines that the same individual who performed the first task must also perform the bound task(s). Similarly, a *role-binding* constraint defines that bound tasks must be performed by members of the same role but not necessarily by the same individual.

### 1.1 Motivation

As outlined above, entailment constraints are an important means to assist the specification of business processes and control their execution. Yet, the runtime enforcement of entailment constraints in distributed SOA business processes is a complex task, and currently there is still a lack of straightforward solutions to achieve this task. This complexity arises from the fact that the tasks of distributed business processes are performed on independent, loosely coupled nodes in a network. One of the advantages of loosely coupled systems is that the different nodes (i.e. services) can execute their tasks independently of other nodes. However, the enforcement of entailment constraints in a distributed system often requires knowledge that is not available to a single node.

Moreover, to enforce access control policies in a software system, the resulting policy models must also be mapped to the implementation level. To account for different platforms and implementation styles, it is important to first establish the enforcement on a generic and conceptual level, in order to map it to concrete platforms (e.g., SOA, as in our case).

Evidently, enforcement of RBAC policies and constraints has an impact on the execution time of business processes. Depending on the complexity of the constraints and the amount of data that needs to be evaluated, the impact will be more or less severe. While the theory behind RBAC and entailment constraints in business processes has been intensively studied in the past, less attention has been devoted to the runtime enforcement, including performance impacts, of such constraints.

With respect to the rapidly increasing importance of process-aware information systems, the correct and efficient implementation of consistency checks in these systems is an important issue. Therefore, the runtime performance needs to be evaluated thoroughly in order to ensure the efficient execution of business processes that are subject to access constraints.

### 1.2 Approach synopsis

This paper builds on our previous work from [14], [21]. In [14], we presented a generic approach for the specification of process-related RBAC models including a corresponding UML extension (see also Sections 2, 3). In [21], we discussed an approach for identity and access management in a SOA context. However, while the enforcement of entailment constraints in a distributed system is a very complex task (see discussion in Section 1.1), neither [14] nor [21] address this important issue. In this paper, we integrate the approaches from [14], [21] and provide multiple novel contributions. In particular, we present an integrated, model-driven approach for the definition and enforcement of RBAC-related entailment constraints in distributed SOA business processes. We extend our textual DSL from [21] with language primitives for the specification of entailment constraints. Furthermore, we significantly extended our implementation and provide an extensive performance evaluation of our solution.

In general, distributed business processes involve stakeholders with different background and expertise. A technical RBAC model may be well-suited for software architects and developers, but for non-technical domain experts an abstracted view is desirable. In the context of model-driven development (MDD) [22], [23], [24], a systematic approach for DSL (*domain-specific language*) development has emerged in recent years (see, e.g., [25], [26], [27], [28]). A DSL is a tailor-made (computer) language for a specific problem domain. To ensure compliance between models and software platforms, models defined in a DSL are mapped to code artifacts via automated model-transformations (see, e.g., [29], [30], [31]). In our approach, the use of a DSL for RBAC constraints allows us to abstract from technical details and involve domain experts in the security modeling procedure.

**Fig. 1:**
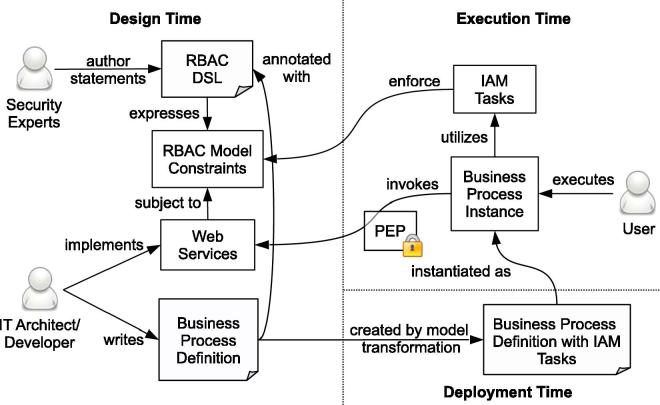
Approach overview.

Fig. 1 depicts a high-level overview of our approach, including the involved stakeholders, system artifacts, and relationships between them. At design time, the security experts author RBAC DSL statements to define the RBAC model and entailment constraints. IT specialists implement Web services and define business processes on top of the services. At deployment time, the process definition files are automatically enriched with tasks for identity and access management (IAM) that conform to the corresponding entailment constraints. The business process is instantiated and executed by human individuals, and the IAM tasks ensure that the process conforms to the constraints defined in the RBAC model. A policy enforcement point (PEP) component intercepts all service invocations to block unauthorized access (see also [21]).

For the sake of platform independence, we model business processes using UML activity diagrams [32]. In particular, we use the *BusinessActivities* extension [14], which enables the definition of process-related RBAC models via extended UML activity models. Based on the generic solution, we discuss a concrete instantiation and show how the approach is mapped to the Web services technology stack, including the Business Process Execution Language for Web services (WS-BPEL) [33].

The remainder of this paper is structured as follows. In Section 2, we present a motivating scenario. Section 3 introduces a generic metamodel for specification of process-related RBAC models including entailment constraints. Section 4 describes the transformation procedure that enriches the process definitions with IAM tasks to enforce runtime-compliance. In Section 5, we present a concrete WS-BPEL-based application of our approach. Implementation-related details are given in Section 6, and in Section 7 we evaluate different aspects of our solution. Section 8 discusses related work, and Section 9 concludes with an outlook for future work.

## 2 Motivating scenario

We illustrate the concepts of this paper based on a scenario taken from the e-health domain. The scenario models the workflow of orthopedic hospitals which treat fractures and other serious injuries. The hospitals are supported by an IT infrastructure organized in a SOA, implemented using Web services. The SOA provides Web services for patient data, connects the departments of different hospitals, and facilitates the routine processes. Because the treatment of patients is a critical task and the personal data constitute sensitive information, security must be ensured and a tailored domain-specific RBAC model needs to be enforced. Task-based entailment constraints in the form of mutual exclusion and binding constraints are a crucial part of the system.

### 2.1 Patient examination business process

**Fig. 2:**
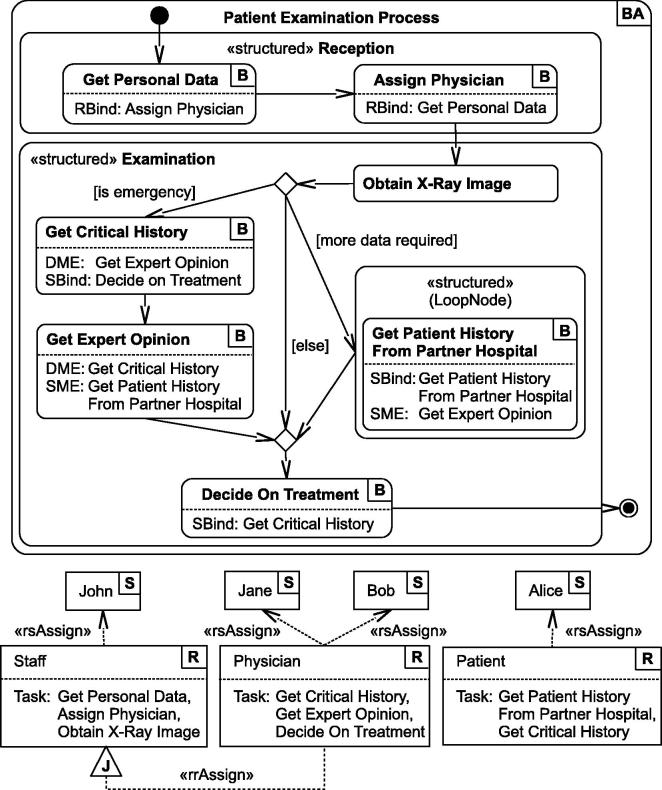
Patient examination scenario modeled as UML business activity.

A core procedure in the hospital is the patient examination, illustrated in Fig. 2 as a *Business Activity* [14] model. We assume that the process is implemented using a business process engine and that the actions (or tasks) represent the invocations of services. The arrows between the actions indicate the control flow of the process. Note that all tasks are backed by technical services, however, part of the tasks are not purely technical but involve some sort of human labor or interaction.

The top part of the figure shows the BusinessActivity model of the process, and the bottom part contains an excerpt of the RBAC definitions that apply to the scenario. We define three types of roles (Staff, Physician, Patient), each with a list of tasks they are permitted to execute, and four subjects (John, Jane, Bob, Alice), each with roles assigned to them. The names of permitted tasks of a role are displayed after the string *“Task:”*. Note, however, that this is only one possible graphical presentation option to display the association between roles and actions (see [14]). Role inheritance hierarchies are modeled using the role-to-role assignment (*rrAssign*) relationship (senior-roles inherit the permissions of junior-roles, e.g., Physician inherits from Staff). The role-to-subject assignment (*rsAssign*) association is used to assign roles to subjects.

The first step in the examination process (see Fig. 2) is to retrieve the personal data of the patient. To demonstrate the cross-organizational character of this scenario, suppose that the patient has never been treated in our example hospital (H1) before, but has already received medical treatment in a partner hospital (H2). Consequently, H1 obtains the patient’s personal data via the Web services of H2. Secondly, the patient is assigned to a physician. After the patient has been assigned, the physician requests an X-ray image from the responsible department. The physician then decides whether additional data are required (e.g., information about similar fractions or injuries in the past). If so, the business process requests historical data from partner hospitals which also participate in the SOA. For privacy reasons, the historical data are only disclosed to the patient herself, and the *Get Patient History* service task has to execute under the role *Patient* (see Fig. 2). Another situation that requires additional data is the case of an emergency. If the emergency demands for immediate surgery, it is important to determine historical data about any critical conditions or diseases that might interfere with the surgery (task *Get Critical History*). To avoid that a single physician takes wrong decisions in an emergency, it is mandatory to get the opinion of a second expert. Finally, the task *Decide On Treatment* completes the examination and triggers the (physical) treatment.

### 2.2 Entailment constraints

In this paper, we support four types of entailment constraints which we briefly discuss in the following. The scenario process in Fig. 2 contains examples for each type of constraint.

• *Static Mutual Exclusion* (SME): The SME constraint between *Get Expert Opinion* and *Get Patient History from Partner Hospital* defines that the two tasks must never be executed by the same subject or role, across all process instances. This constraint is reasonable as we need to explicitly separate the permissions of patients and physicians.
• *Dynamic Mutual Exclusion* (DME): The DME constraint for *Get Critical History* and *Get Expert Opinion* requires that, for each instance of the process, these two tasks are executed by different subjects. This ensures that the treatment decision in an emergency clearly depends on the medical assessment of two individual physicians.
• *Subject Binding* (SBind): An example SBind constraint is the *Get Patient History From Partner Hospital* task, which executes multiple times in a loop. To ensure that each iteration is done by the same subject, the *SBind* attribute reflexively links to the same task. A second subject binding exists between *Get Critical History* and *Decide on Treatment*.
• *Role Binding* (RBind): The process defines a role-binding constraint which demands that the *Get Personal Data* and *Assign Physician* are performed by the same role (although potentially different subjects).

## 3 Generic metamodel for specification of entailment constraints in business processes

This section gives an overview of the generic metamodel for specification of process-related RBAC models including entailment constraints. To provide a self-contained view in this paper, Section 3.1 repeats the core definitions from [14], which form the basis for our approach. In Section 3.2, we introduce the textual RBAC DSL which allows to define entailment constraints in a simple textual syntax and enables a seamless mapping of UML-based process-related RBAC models (see [14]) to the implementation level. The core part of the textual RBAC DSL is based on [21]. For this paper, it has been extended with capabilities for the specification of entailment constraints.

### 3.1 Business activity RBAC models

Definition 1 **Business Activity RBAC Model**

A Business Activity RBAC Model *BRM* = (*E*, *Q*, *D*) where *E* = *S* ∪ *R* ∪ *P*_*T*_ ∪ *P*_*I*_ ∪ *T*_*T*_ ∪ *T*_*I*_ refers to pairwise disjoint sets of the metamodel, *Q* = *rh* ∪ *tra* ∪ *rsa* ∪ *ptd* ∪ *pi* ∪ *ti* ∪ *es* ∪ *er* to mappings that establish relationships, and *D* = *sb* ∪ *rb* ∪ *sme* ∪ *dme* to binding and mutual exclusion constraints, such that:

• For the sets of the metamodel:
  – An element of *S* is called *Subject*. *S* ≠ ∅.
  – An element of *R* is called *Role*. *R* ≠ ∅.
  – An element of *P*_*T*_ is called *Process Type*. *P*_*T*_ ≠ ∅.
  – An element of *P*_*I*_ is called *Process Instance*.
  – An element of *T*_*T*_ is called *Task Type*. *T*_*T*_ ≠ ∅.
  – An element of *T*_*I*_ is called *Task Instance*.

In the list below, we iteratively define the partial mappings of the Business Activity RBAC Model and provide corresponding formalizations (P refers to the power set, for further details see [14]):

1. The mapping rh:R↦P(R) is called **role hierarchy**. For *rh*(*r*_*s*_) = *R*_*j*_ we call *r*_*s*_ *senior role* and *R*_*j*_ the set of direct *junior roles*. The transitive closure *rh*^∗^ defines the inheritance in the role hierarchy such that ${\mathrm{rh}}^{*}(r_{s})=R_{j^{*}}$ includes all direct and transitive junior roles that the senior role *r*_*s*_ inherits from. The role hierarchy is cycle-free, i.e. for each *r* ∈ *R*:*rh*^∗^(*r*) ∩ {*r*} = ∅.
2. The mapping $\mathrm{tra}:R\;\mapsto \;P(T_{T})$ is called **task-to-role assignment**. For *tra*(*r*) = *T*_*r*_ we call *r* ∈ *R role* and *T*_*r*_ ⊆ *T*_*T*_ is called the set of *tasks assigned to r*. The mapping ${\mathrm{tra}}^{-1}:T_{T}\;\mapsto \;P(R)$ returns the set of roles a task is assigned to (the set of roles owning a task).
  This assignment implies a mapping **task ownership** $\mathrm{town}:R\;\mapsto \;P(T_{T})$, such that for each role *r* ∈ *R* the tasks inherited from its junior roles are included, i.e. $\mathrm{town}(r)={⋃}_{r_{\mathrm{inh}}\in {\mathrm{rh}}^{*}(r)}\mathrm{tra}(r_{\mathrm{inh}})\cup \mathrm{tra}(r)$. The mapping ${\mathrm{town}}^{-1}:T_{T}\;\mapsto \;P(R)$ returns the set of roles a task is assigned to (directly or transitively via a role hierarchy).
3. The mapping rsa:S↦P(R) is called **role-to-subject assignment**. For *rsa*(*s*) = *R*_*s*_ we call *s* ∈ *S subject* and *R*_*s*_ ⊆ *R* the set of *roles assigned to this subject* (the set of roles owned by *s*). The mapping ${\mathrm{rsa}}^{-1}:R\;\mapsto \;P(S)$ returns all subjects assigned to a role (the set of subjects owning a role).
  This assignment implies a mapping **role ownership** rown:S↦P(R), such that for each subject *s* ∈ *S* all direct and inherited roles are included, i.e. *rown*(*s*) = ⋃ _*r*∈*rsa*(*s*)_ *rh*^∗^(*r*) ∪ *rsa*(*s*). The mapping ${\mathrm{rown}}^{-1}:R\;\mapsto \;P(S)$ returns all subjects assigned to a role (directy or transitively via a role hierarchy).
4. The mapping $\mathrm{ptd}:P_{T}\;\mapsto \;P(T_{T})$ is called **process type definition**. For $\mathrm{ptd}(p_{T})=T_{p_{T}}$ we call *p*_*T*_ ∈  *P*_*T*_ process type and $T_{p_{T}}\subseteq T_{T}$ the set of task types associated with *p*_*T*_.
5. The mapping $\mathrm{pi}:P_{T}\;\mapsto \;P(P_{I})$ is called **process instantiation**. For *pi*(*p*_*T*_) = *P*_*i*_ we call *p*_*T*_ ∈ *P*_*T*_ *process type* and *P*_*i*_ ⊆ *P*_*I*_ the set of process instances instantiated from process type *p*_*T*_.
6. The mapping $\mathrm{ti}:(T_{T}\times P_{I})\;\mapsto \;P(T_{I})$ is called **task instantiation**. For *ti*(*t*_*T*_,*p*_*I*_) = *T*_*i*_ we call *T*_*i*_  ⊆ *T*_*I*_ set of *task instances*, *t*_*T*_ ∈ *T*_*T*_ is called *task type* and *p*_*I*_ ∈ *P*_*I*_ is called *process instance*.
7. Because role-to-subject assignment is a many-to-many relation (see Def.1.3), more than one subject may be able to execute instances of a certain task type. The mapping *es*:*T*_*I*_ ↦ *S* is called **executing-subject** mapping. For *es*(*t*) = *s* we call *s* ∈ *S* the *executing subject* and *t* ∈ *T*_*I*_ is called *executed task instance*.
8. Via the role hierarchy, different roles may posses the permission to perform a certain task type (see Def.1.1 and Def.1.2). The mapping *er*:*T*_*I*_ ↦ *R* is called **executing-role** mapping. For *er*(*t*) = *r* we call *r* ∈ *R* the *executing role* and *t* ∈ *T*_*I*_ is called *executed task instance*.
9. The mapping $\mathrm{sb}:T_{T}\;\mapsto \;P(T_{T})$ is called **subject-binding**. For *sb*(*t*_1_) = *T*_*sb*_ we call *t*_1_ ∈ *T*_*T*_ the *subject binding task* and *T*_*sb*_ ⊆ *T*_*T*_ the set of *subject bound tasks*.
10. The mapping $\mathrm{rb}:T_{T}\;\mapsto \;P(T_{T})$ is called **role-binding**. For *rb*(*t*_1_) = *T*_*rb*_ we call *t*_1_ ∈ *T*_*T*_ the *role binding task* and *T*_*rb*_ ⊆ *T*_*T*_ the set of *role bound tasks*.
11. The mapping $\mathrm{sme}:T_{T}\;\mapsto \;P(T_{T})$ is called **static mutual exclusion**. For *sme*(*t*_1_) = *T*_*sme*_ with *T*_*sme*_  ⊆ *T*_*T*_ we call each pair *t*_1_ ∈ *T*_*T*_ and *t*_*x*_ ∈ *T*_*sme*_ *statically mutual exclusive tasks*.
12. The mapping $\mathrm{dme}:T_{T}\;\mapsto \;P(T_{T})$ is called **dynamic mutual exclusion**. For *dme*(*t*_1_) = *T*_*dme*_ with *T*_*dme*_  ⊆ *T*_*T*_ we call each pair *t*_1_ ∈ *T*_*T*_ and *t*_*x*_ ∈*T*_*dme*_ *dynamically mutual exclusive tasks*.

### 3.2 RBAC modeling for business processes

**Fig. 3:**
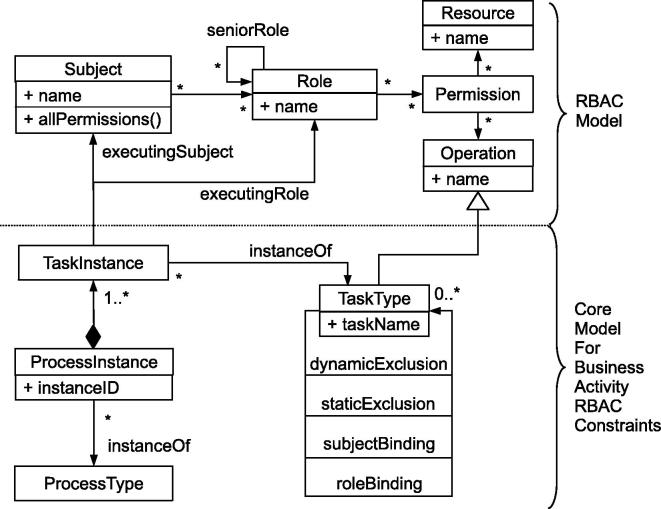
Excerpt of RBAC metamodel and business activity metamodel.

Fig. 3 depicts the core RBAC metamodel and its connection with the core elements of the BusinessActivity metamodel. In particular, Fig. 3 outlines how we extended our DSL from [21] to include process-related RBAC entailment constraints (see [14]). The different model elements are described below.

A ProcessInstance has a unique instanceID, a ProcessType, and is composed of multiple TaskInstance objects which are again instances of a certain TaskType. The class TaskType has a name and four reflexive associations that define mutual exclusion and binding constraints. Subjects are identified by a name attribute and are associated with an arbitrary number of Roles, which are themselves associated with Permissions to execute certain Operations. A TaskType in the BusinessActivity metamodel corresponds to an Operation in the RBAC metamodel. Roles may inherit permissions from other roles (association seniorRole). In our approach, we directly associate Web service instances with Resources. That is, a subject that attempts to invoke a Web service operation *op* on a service resource *res* must be associated with a role that holds a permission to execute *op* on *res*. A detailed description of the BusinessActivity metamodel and corresponding OCL (Object Constraint Language) constraints can be found in [14]. We utilize the core parts of this model and focus on the mapping of the RBAC constraints to a textual DSL and to business process execution platforms, as illustrated for WS-BPEL in Section 5.

### 3.3 RBAC DSL statements

Our RBAC DSL is implemented as an embedded DSL [27] and is based on the scripting language *Ruby* as host programming language. We now briefly discuss how the model elements are mapped to language constructs provided by the DSL (see also Section 3.1 and Fig. 3). Table 1 lists the basic DSL statements (left column) and the corresponding effect (right column). In the table, keywords of the DSL syntax are printed in **bold typewriter** font, and placeholders for custom (scenario-specific) expressions are printed in *italics*.

The RBAC DSL statements RESOURCE, OPERATION, SUBJECT and ROLE are used to create resources, operations, subjects and roles with the respective *name* and optional *description* attributes. ASSIGN creates an association between a subject and a role. INHERIT takes two parameters, a junior-role and a senior-role name, and causes the senior-role to inherit all permissions of the junior-role. PERMIT expresses the permission for a role to execute a certain operation on a resource. DME and SME allow the specification of dynamically or statically mutual exclusive operations. Using RBIND and SBIND, two operations are subjected to role-binding or subject-binding constraints. Finally, the TASK statement is used to establish a mapping from our RBAC DSL to implementation level artifacts. More precisely, operations are mapped to concrete WS-BPEL tasks (see Section 5.2). The complete access control configuration for the patient examination scenario, expressed via RBAC DSL statements, is printed in Appendix A.

## 4 Model transformations of process definitions for runtime constraint enforcement

To enforce the RBAC constraints at runtime, the business process needs to follow a special procedure. If the process executes a secured task, it needs to provide a valid authentication token for the active user. For instance, this token contains information which subject (e.g., “Jane”) executes an operation, and under which role (e.g. “Staff”) this individual operates. In this section, we discuss our approach for automatically obtaining these authentication tokens to enforce security at runtime.

**Fig. 4:**
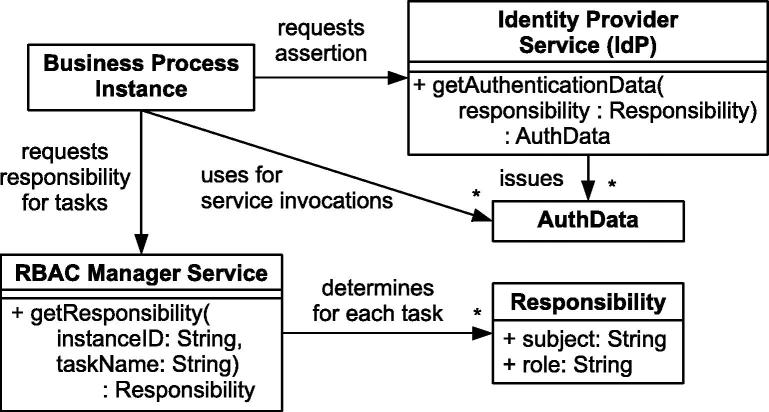
Relationship between business process instance and security enforcement artifacts.

Fig. 4 illustrates which artifacts are utilized by the instances of the business process. We follow the concepts of the SAML framework [34] and provide the authentication data with the aid of an Identity Provider (*IdP*) service. An IdP is a service provider that maintains identity information for users and provides user authentication to other services. The IdP is a reusable standard component; its sole responsibility is to authenticate the user and to issue an *AuthData* document which asserts the user’s identity (subject and role). As such, the IdP has no knowledge about the process structure and RBAC constraints. Hence, we utilize the decoupled RBAC Manager Service which keeps track of the state of the process instances. The RBAC Manager Service knows the process structure and decides, based on the RBAC constraints, which subject or role is responsible for the next task (see also [19]).

Combining the functionality of getResponsibility and getAuthenticationData (see Fig. 4) constitutes the core protocol for obtaining authentication tokens that enable the enforcement of task-based entailment constraints in a BusinessActivity. This recurring protocol is executed for each secured task; hence, it need not be implemented manually, but should ideally be generated automatically on top of the business process model that is defined by the developer. We therefore aim at providing automatic transformations to convert the domain-specific extensions for mutual exclusion and binding constraints in BusinessActivity models into regular activity models which perform the required IAM tasks. This transformation is required as an intermediate step towards the generation of corresponding definitions that are directly deployable and executable (e.g., by WS-BPEL engines). In the following, we describe the transformation procedure in detail and discuss different implementation and runtime aspects.

### 4.1 Model transformations to enforce mutual exclusion constraints

**Fig. 5:**
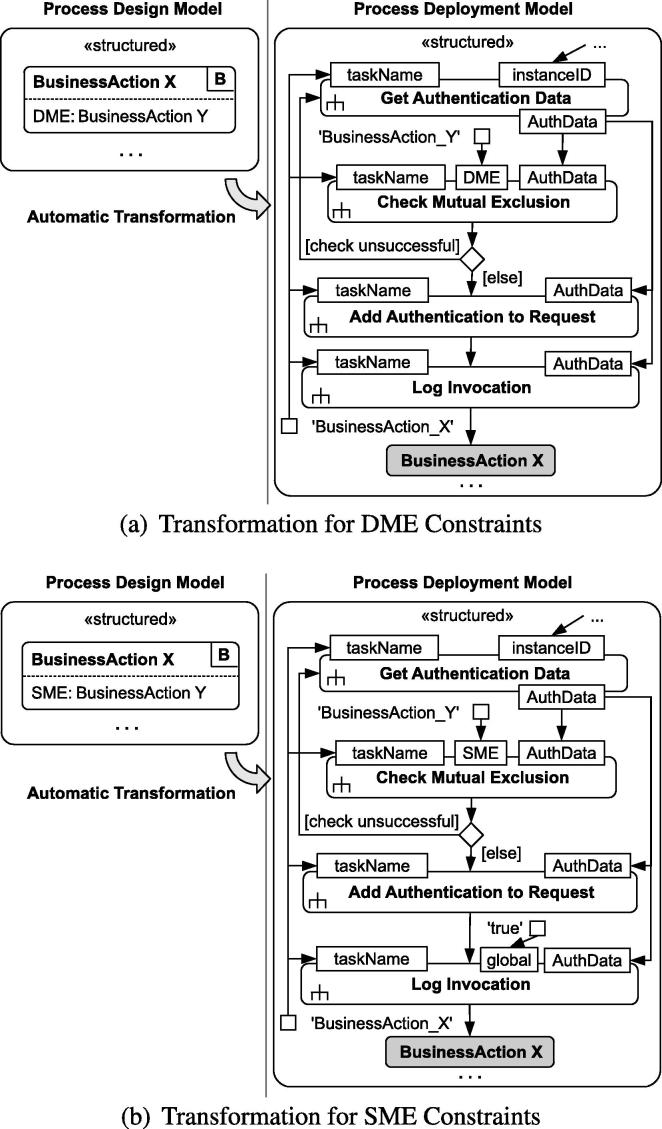
Process transformations to enforce mutual exclusion constraints.

Here we discuss the detailed procedure for runtime enforcement of mutual exclusion constraints in the form of DME and SME tasks. We propose an approach for transforming design-time BusinessActivity models into deployable standard activity models that comply with this procedure. The transformations for enforcing mutual exclusion constraints are illustrated in Fig. 5. Tasks representing invocations to external Web services are printed in gray, while structured activities and tasks with local processing logic are depicted with a white background.

The transformed activity models with mutual exclusion constraints in Fig. 5 contain four additional tasks. All four tasks are UML *CallBehaviorAction*s [32] (indicated by the rake-style symbol) which consist of multiple sub-tasks. The internal processing logic depends on the concrete target platform; later in Section 5.1 we discuss the detailed logic for WS-BPEL.

The task *Get Authentication Data* invokes the IdP service to obtain the authentication data token (*AuthData*) to be used for later invocation of the BusinessAction. The second inserted task is *Check Mutual Exclusion*, which is responsible for checking whether the provided authentication data are valid with respect to the mutual exclusion constraint. A UML value pin [32] holding the name of the corresponding task provides the input for the pin *DME* (Fig. 5a) or the pin *SME* (Fig. 5b), respectively. Additionally, the *Check Mutual Exclusion* task receives as input the name of the task to-be-executed (*taskName*, which is known from the original process definition), and the *AuthData* (received from the IdP service). The decision node is used to determine whether *Check Mutual Exclusion* has returned a successful result. If the result is unsuccessful (i.e., a constraint violation has been detected) the control flow points back to *Get Authentication Data* to ask the IdP again for a valid authentication data token. Otherwise, if the result is successful, the task *Add Authentication to Request* appends the user credentials in *AuthData* to the request message for the target Web service operation. The fourth inserted task is *Log Invocation*, which adds a new log record that holds the name of the task (*taskName*) and the *AuthData* of the authenticated user. The input pin *global* determines whether the log entry is stored in a local variable of the process instance (value *null*) or in a global variable accessible from all process instances (value ‘*true*’).

### 4.2 Model transformations to enforce binding constraints

**Fig. 6:**
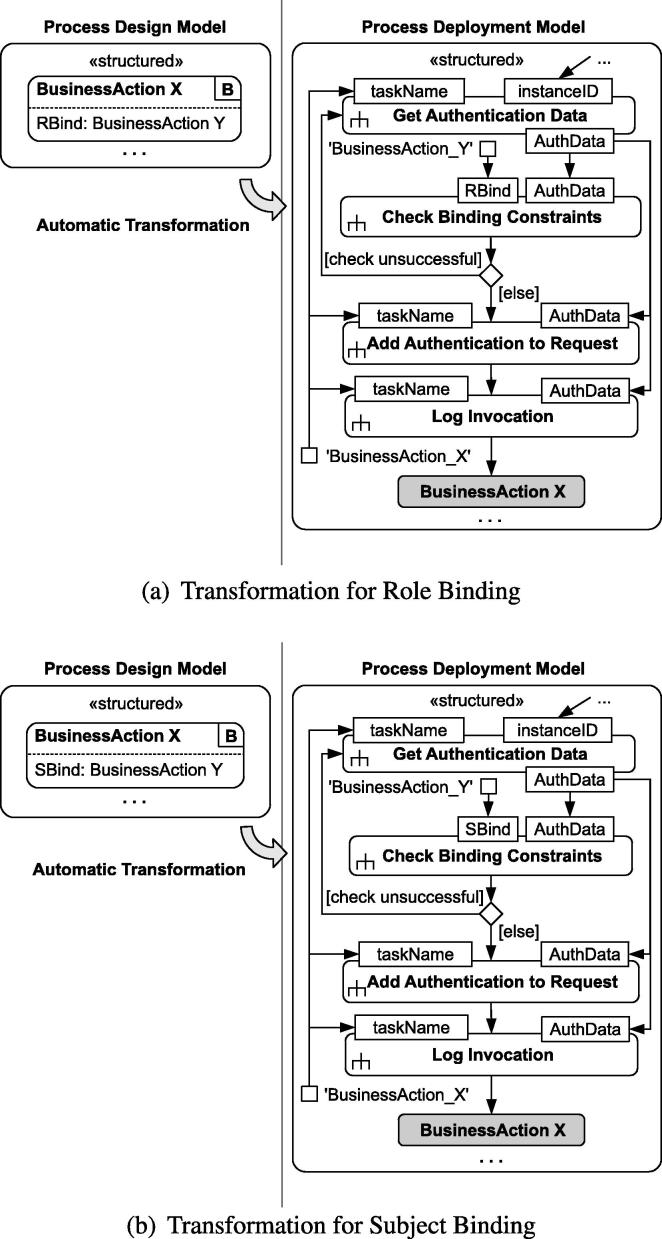
Process transformations to enforce binding constraints.

The approach for transforming binding constraints in BusinessActions (illustrated in Fig. 6) is similar to the transformation for mutual exclusion constraints presented in Section 4.1. The transformed process model first requests authentication data from the IdP service. The task *Check Binding Constraints* then checks the resulting *AuthData* with respect to role-bindings (*RBind*, Fig. 6a) and subject-bindings (*SBind*, Fig. 6b). The process asks for new user credentials and repeats the procedure if the binding constraint is not fulfilled.

Note that the entailment constraints are checked directly inside the process, not by the IdP service. Even though the AuthData (subject, role) obtained from the IdP is trusted and assumed to properly represent the user executing the process, the AuthData may be invalid with respect to entailment constraints. Hence, the branch “check unsuccesful” indicates that the process instance asks for a different user to login and execute the task. As the log of previous tasks is stored locally by each process instance (except for SME constraints, where log entries are also stored globally), the *Check Binding* and *Check Mutual Exclusion* tasks are required directly inside the process logic and are not outsourced to external services. This approach is able to deal with deadlock situations (evaluated in Section 7.2).

In certain deployments, the platform providers (e.g., hospital management) may be interested in tracking failed authorizations. For brevity, such mechanisms are not included in Fig. 5, Fig. 6, but extending the approach with notifications is straight-forward.

### 4.3 Transformation rules for combining multiple constraints

So far, the transformation rules for the four different types of entailment constraints in BusinessActivities (role-binding, subject-binding, SME, DME) have been discussed in isolation. However, as the scenario in Section 2 illustrates, Business-Actions can possibly be associated with multiple constraints (e.g., *Get Critical History*). Therefore, we need to analyze how the transformation rules can be combined while still maintaining the constraints’ semantics. A simple approach would be to successively apply the atomic transformations for each BusinessAction and each of the constraints associated with it. However, this approach is not suited and may lead to incorrect results. For instance, if we consider the task *Get Critical History* with the associated DME and SBind constraints, the process might end up requesting the authentication data twice, which is not desired. Therefore, multiple constraints belonging to the same task are always considered as a single unit (see also [19]).

**Fig. 7:**
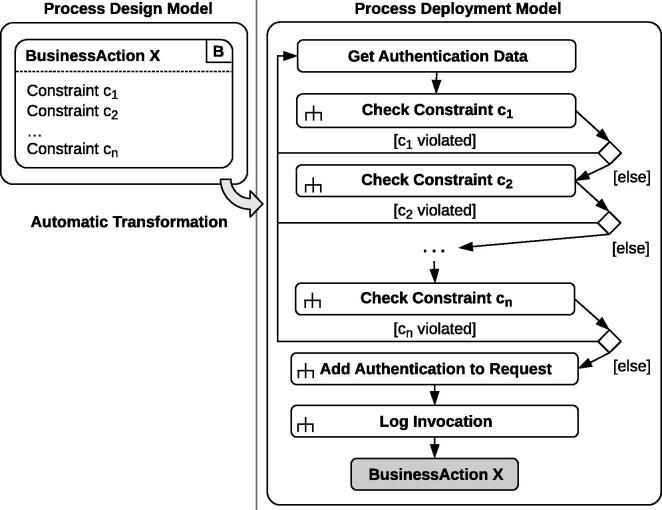
Generic transformation template for business action with multiple constraints.

Fig. 7 depicts the transformation template for a generic sample BusinessAction *X* with multiple constraints *c*_1_, *c*_2_, … , *c*_*n*_.

## 5 Application to SOA and WS-BPEL

This section discusses details of the process transformation from Section 4 and illustrates how the approach is applied to SOA, particularly WS-BPEL and the Web services framework.

### 5.1 Supporting tasks for IAM enforcement in WS-BPEL

In the following we discuss the internal logic of the five supporting IAM tasks used in the transformed activity models for the enforcement of mutual exclusion (Section 4.1) and binding constraints (Section 4.2).

**Fig. 8:**
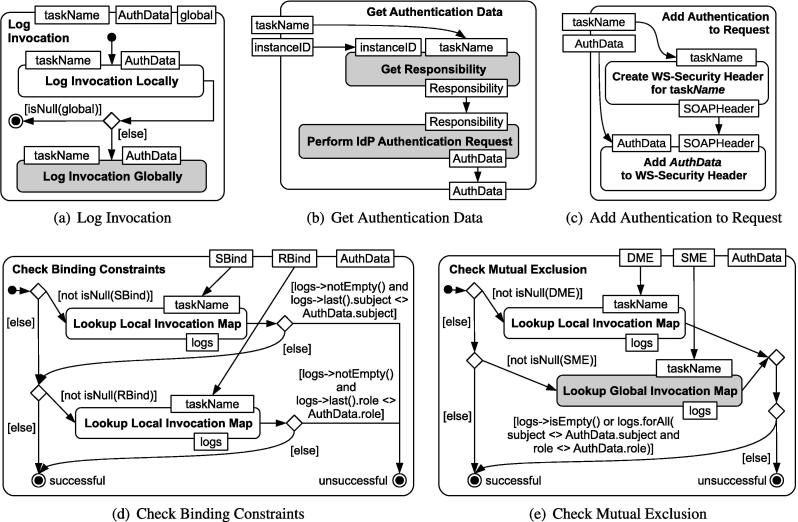
Supporting tasks for IAM enforcement in WS-BPEL.

Task **Log Invocation**: In general, process-related RBAC constraints rely on knowledge about historical task executions (see also [14]). Therefore, a mechanism is required to store data about previous service invocations. One conceivable approach is that the process execution engine keeps track of the invocation history. To that end, invocation data can be stored either in a local variable of the process instance (for DME constraints) or in a global variable that is accessible from all process instances (for SME constraints). Unfortunately, WS-BPEL does not support global variables, but we can overcome this issue by using an external logging Web service. Fig. 8a shows the *Log Invocation* activity, which stores data about service calls, including the name of the invocation and the *AuthData* of the user under which the process executes. The invocation is first stored in a local array variable of WS-BPEL. If the input pin named *global* is not null, the data is also stored with the external logging service (*Log Invocation Globally*). Currently, our framework relies on a central logging service. As part of our future work, we tackle advanced challenges such as privacy, and timing issues that come with decentralized logging.

Task **Get Authentication Data**: This supporting IAM task is used to obtain authentication tokens, see Fig. 8b. The identifier of the affected process task is provided as a parameter *taskName*. For instance, in the case of WS-BPEL, the name attribute of the corresponding invoke statement can be used to determine this value. As outlined in Section 4, the procedure is split up between the RBAC Manager service and the IdP. First, the invocation *Get Responsibility* asks the RBAC Manager for the role or subject responsible for executing the next task. All combinations of values are possible, i.e., either subject or role, or both, or none of the two may be specified. The subject/role responsibility information is used to execute an *IdP Authentication Request*. The authentication method performed by the IdP is transparent; for instance, it may perform smartcard based authentication or ask for username and password. The *AuthData* output pin provided by this invocation contains the definite subject and role name of the user.

Task **Add Authentication to Request**: The activity in Fig. 8c illustrates how authentication data are appended to the invocation of Business Actions. First, the *AuthData* information is used to request a SAML assertion from the IdP service. This token contains the subject and role with a trusted signature that ensures the integrity of the assertion content. The assertion is then added to the request message for the target service operation (the name is specified via the input pin *taskName*) using the SOAP header mechanism [35] (SOAP is the communication protocol used by Web services). Note that this activity leaves room for optimization. If many tasks in the process are executed by the same subject and role, it is advantageous to cache and reuse the SAML tokens in a local variable of the process instance. However, caching security tokens carries the risk of inconsistencies if the RBAC policies change.

Task **Check Binding Constraints**: Fig. 8d contains the activity *Check Binding Constraints*, whose internal logic is to check the logged invocations with role-binding and subject-binding against the *AuthData* information. If the *SBind* parameter is set, the activity looks up the last corresponding log entry (the *taskName* of the log entry needs to be equal to *SBind*) in the local invocation map of the WS-BPEL process instance. If the returned array (named *logs*) is not empty, then the subject stored in the last log entry needs to be identical to the subject in *AuthData*. Analogously, if the *RBind* parameter is set, then the role of the last log entry with *taskName* equal to *RBind* must be equal to the role in *AuthData*. If and only if all conditions hold true, the activity returns a success status.

Task **Check Mutual Exclusion**: Similarly, the *Check Mutual Exclusion* activity in Fig. 8e uses the log data to check the *AuthData* against the previously performed invocations. If the input parameter *DME* is set, WS-BPEL looks up the log entries from the local invocation map. Otherwise, if an *SME* parameter is provided, the corresponding logs are received from the external logging service (global invocation map). The activity returns a successful result if either the *logs* sequence is empty or all log entries have a different subject and role than the given *AuthData*. Due to the possibly large number of entries in the *logs* sequence, it is crucial where these conditions are evaluated (by the process or the logging service directly). To avoid transmitting log data over the network, we recommend the implementation variant in which the logging service itself validates the conditions. To that end, *AuthData* is sent along with the request to the logging service and the service returns a boolean result indicating whether the constraints are satisfied.

### 5.2 RBAC DSL integration with WS-BPEL

The TASK statement of the RBAC DSL realizes a mapping from operations to concrete WS-BPEL tasks (invoke activities). This corresponds to the model in Fig. 3, where TaskType in the Business Activities metamodel is mapped to Operation in the RBAC metamodel. Using this mapping, we are able to automatically apply all Business Activity entailment constraints to the corresponding WS-BPEL invoke activities.

In our approach, WS-BPEL invoke activities are constrained using specialized DSL statements. The DSL uses the extension mechanism of WS-BPEL and introduces new XML attributes rbac:dme, rbac:sme, rbac:sbind and rbac:rbind (the prefix rbac refers to the XML namespace these attributes are part of). These attributes are then directly annotated to the invoke activities in WS-BPEL. Table 2 illustrates how the relevant RBAC DSL statements are mapped to the corresponding WS-BPEL DSL statements. For instance, the DME statement is mapped to a rbac:dme attribute. The parameters of the DSL statements in Table 2 refer to the task types defined using the TASK statement (see Section 3.3). Note that these rbac:∗ attributes can be multi-valued. That is, multiple values can be separated by commas. For example, a task that is dynamically mutual exclusive to *task*1 and *task*2 can be rbac:dme = "task1,task2" attribute.

### 5.3 Automatic transformation of WS-BPEL definition

At deployment time, the business process model is automatically transformed to ensure correct enforcement of identity and access control policies at runtime. The transformation can happen on different abstraction levels, either based on the platform-independent model (PIM) or on the platform-specific model (PSM) (see, e.g., [36]). On the PIM level, model transformation languages such as *Query/View/Transformation* (QVT) [37] can be used to perform UML-to-UML transformation of process activity models. Our approach proposes a transformation directly on the PSM model, i.e., the WS-BPEL process definition file.

Algorithm 1 **WS-BPEL Transformation Algorithm**

**Table:** 

**Input**: WS-BPEL document *bpel*, Fragment Templates *tmpl*
**Output**: transformed WS-BPEL document
1: add < import ../>, < partnerLink ../>, and < variable ../> statements to *bpel*
2: add < assign ../> statements to initialize *ProcessInstanceID* and *InvocationLogs* variables
3: **for all***bpel*//invoke as *inv***do**
4: **if***inv*/@rbac:*then
5: *authInvoke* ← create < invoke ../> for operation *getAuthenticationData* and partnerLink *IdP*
6: *constraintChecks* ← ∅
7: **for all***inv*/@rbac:* as *constraint***do**
8: *tasks*← split value of *constraint* by commas
9: **for all***tasks* as *task***do**
10: *check* ← create < if>..</if > which checks outcome of *authInvoke* for RBAC entailment constraint *constraint* and task *task*
11: *constraintChecks* ← *constraintChecks* ∪ *check*
12: **end for**
13: **end for**
14: *enforcementBlock* ← wrap sequence *authInvoke*∥*constraintChecks* in new < while>..</while > block
15: insert *enforcementBlock* before *inv*
16: **if***inv*/@rbac:sme**then**
17: *logInvoke*← create < invoke ../> for operation *logInvocation* via partnerLink *LoggingService*
18: insert *logInvoke* after *inv*
19: **end if**
20: **end if**
21: **end for**

Algorithm 1 gives a simplified overview of which WS-BPEL code fragments are injected, and where. Variable names are printed in *italics*, and XML markup and XPath expressions are in typewriter font. The input is a WS-BPEL document *bpel* with security annotations. Firstly, various required documents (e.g. the XSD files of SAML and WS-Security) need to be imported into the WS-BPEL process using import statements. Then the partnerLink declarations for the needed services (such as the IdP service) are added to *bpel*, and variable declarations are created (e.g. input/output variables for getAuthenticationData operations). Using assign statements, some variables (such as ProcessInstanceID) are initialized. Next, the algorithm loops over all invoke elements that have an attribute from the rbac namespace assigned (e.g. rbac:rbind or rbac:dme). For every matching invoke several WS-BPEL code injections and transformations have to be conducted. Firstly, an invoke statement (authInvoke) is created. At runtime, this statement calls the IdP’s getAuthenticationData operation. Next, an empty set (constraintChecks) is created. Afterwards, the algorithm iterates over all constraints (e.g. rbac:sbind) that have been defined for this particular invoke statement. The values of every constraint are split by commas. For instance, in the case of an rbac:dme = "task1,task2" annotation, constraint is rbac:dme and tasks is a set with two elements (task1 and task2). For every task an if-block (check) is created. At runtime, this if-block checks, if there is a violation of the entailment constraint constraint regarding another task task. Every check added to the set constraintChecks. Next, a new  < while>..</while>-block (enforcementBlock) is created. This block envelopes the previously created authInvoke statement and all checks contained in constraintChecks. Finally, this enforcementBlock is inserted directly before the secured invoke statement. Just in case the latter is also annotated using a rbac:sme attribute, an additional invocation is injected right after the actual invoke element. This one calls the logInvocation operation via the LoggingService PartnerLink.

## 6 Implementation

In this section, we discuss our prototype implementation of the proposed approach. The implementation is integrated in the SeCoS^1^ (*Se*cure *Co*llaboration in *S*ervice-based systems) framework. This section is divided into four parts: firstly, we outline the architecture of the system and the relationship between the individual services and components in Section 6.1; secondly, the SAML-based SSO mechanism is described in Section 6.2; in Section 6.3 we present the algorithm for automatic transformation of WS-BPEL definitions containing security annotations from our DSL; finally, Section 6.4 discusses the implementation for checking constraints over the log data.

### 6.1 System architecture

**Fig. 9:**
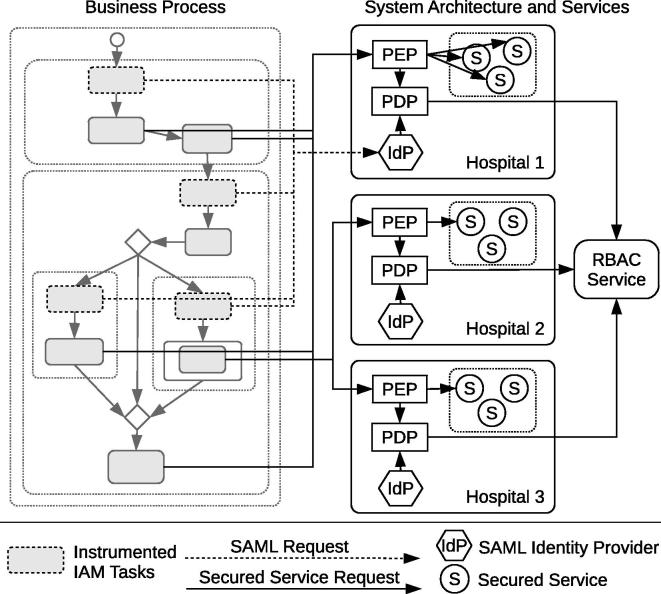
Example process in system architecture.

Fig. 9 sketches the high-level architecture and relationships between the example process and the system components.

The patient examination scenario from Section 2 is implemented using WS-BPEL and deployed in a Glassfish^2^ server. The scenario involves three hospitals, which host the protected services for patient management and examination. All service invocations are routed through a Policy Enforcement Point (**PEP**), which acts as a central security gateway, intercepts every incoming service request and either allows or disallows its invocation. It is important that the PEP operates transparently and as close to the protected resources (i.e., services) as possible. Using the Java API for XML Web services (JAX-WS), the PEP has been implemented as a SOAP message handler (interface SOAPHandler). This handler can be plugged into the Web service’s runtime engine in a straightforward manner. Once activated, the interceptor is able to inspect and modify inbound and outbound SOAP messages and to deny service invocations.

Each hospital runs a SAML **IdP** service, which is used to issue the SAML assertions that are required in the WS-BPEL process. The IdP’s responsibility is twofold: firstly, it authenticates users; secondly, the IdP assures the identity of a subject and its associated attributes (e.g., roles) by issuing a SAML assertion SOAP header which is used in subsequent service invocations. For the sake of an easy integration into the given system environment, we decided to use the JAX-WS API for implementing the Login Web service. This SOAP Web service offers a login method. It requires a username/password pair and returns a SAML assertion. Internally, we utilize the Java Architecture for XML Binding (JAXB) for parsing and creating SAML assertions. Additionally, the Apache XML Security for Java^3^ library is used for digitally signing XML documents (i.e., the SAML assertions).

The actual decision whether an invocation should be prevented or not is typically delegated to another entity, the Policy Decision Point (**PDP**). When deciding over the access to a service resource the PDP has to make sure that the subject attempting to access the resource has the permission to do so. This decision is based on the policy information stored in the RBAC repository (which is based on the DSL statements authored by domain experts). In our implementation, the core functionality of the PDP is embedded into the RBAC DSL (see Section 3.2). That is, the DSL offers an access method that can be used to determine whether the requesting subject is permitted to access the target resource (service) under the specified context and role (see Fig. 9). In order to make this functionality accessible to the outside of the DSL’s interpreter, we developed a RESTful Web service, that bridges HTTP requests to the interpreter. More precisely, the PDP service uses the Bean Scripting Framework (BSF)^4^ to access the interpreter. The Java API for RESTful Web Services (JAX-RS) is used to realize the PDP service’s RESTful Web interface.

### 6.2 SAML-based single sign-on

**Fig. 10:**
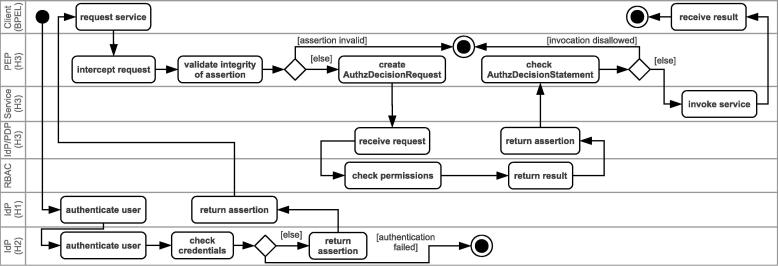
Identity and access control enforcement procedure.

Fig. 10 depicts an example of the Identity and Access Control enforcement procedure modeled in UML. To illustrate the SSO aspect of the scenario, we assume that a patient with subject name “Alice” (cf. Fig. 3), who is registered in hospital 2 (H2), is examined in hospital 1 (H1) and requests her patient history from previous examinations in hospital 3 (H3). The procedure is initiated by the WS-BPEL process which requests the execution of a protected Web service.

Prior to issuing the actual service request, the user has to authenticate using the SAML IdP. The IdP queries the user database to validate the credentials provided by the client. As the credentials of user *Alice* are not stored in the DB of H1, the IdP contacts the IdP of H2, which validates the credentials.

If the user credentials could not be validated, the process is terminated prematurely and a SOAP fault message is returned. In our example scenario, the business process receives the fault message and activates corresponding WS-BPEL fault handlers. Otherwise, if the credentials are valid, the IdP creates a signed assertion similar to the one shown in Listing 1 and passes it back to the WS-BPEL process (see Fig. 10). The business process attaches the assertion to the actual service request.

The example SAML assertion in Listing 1 illustrates the information that is encapsulated in the header token when the scenario process invokes the getPatientHistory operation of the patient Web service of H3. The assertion states that the subject named Alice, which has been successfully authenticated by the IdP of the hospital denoted by the Issuer element (H2), is allowed to use the role staff in the context default. The included XML signature element ensures the integrity of the assertion, i.e., that the assertion content indeed originates from the issuing IdP (H2) and has not been modified in any way. When the PEP of H3 intercepts the service invocation with the SAML SOAP header, its first task is to verify the integrity of the assertion. The signature verification requires the public key of the IdP that signed the assertion; this key is directly requested from the corresponding IdP (under http://h2.com/IdP) using SAML Metadata [38]. Our implementation uses the Apache XML Security for Java library to conduct the signature verification.

After the PEP of H3 has verified the message integrity, it needs to determine whether the subject is authorized to access the requested service operation. This is achieved by the PDP service of H3 that allows the PEP to post a SAML Authorization Decision Query. The PDP answers this query by returning an assertion containing a SAML Authorization Decision Statement. Listing 2 shows an example SAML assertion which informs the PEP that our staff member is allowed to invoke the action (operation) getPersonalData of the resource (Web service) http://h1.com/patient. Analogously to the IdP service, we also used the JAX-WS API to implement the SOAP-based interface of the PDP service. The PDP offers the method query, which takes an Authorization Decision Query message as argument and returns an Authorization Decision Statement. Again, we leverage JAXB for parsing the SAML documents.

### 6.3 Automatic transformation of WS-BPEL definition

Since both WS-BPEL and SAML are XML based standards, we are able to reuse and utilize the broad line-up of existing XML tooling. The transformation procedure of WS-BPEL process definitions is hence based on XSLT (Extensible Stylesheet Language Transformations) [39], a language for arbitrary transformation and enrichment of XML documents.

**Fig. 11:**
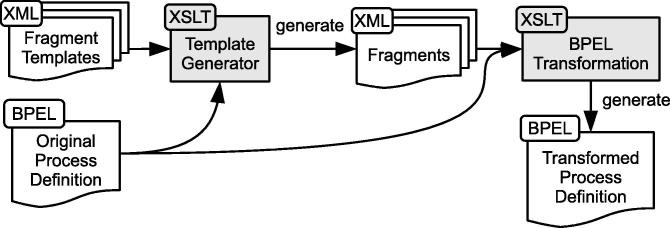
Artifacts of the transformation process.

In general, the original WS-BPEL process is transformed by enriching the process definition file with code fragments that perform the IAM tasks (cf. Section 5.1). In principle, these fragments are generic and static, i.e., for arbitrary WS-BPEL processes nearly the same fragments can be injected. However, some fragments contain volatile elements that are specific to every single WS-BPEL process. As these fragments need to be adapted to fit a specific WS-BPEL process, we propose a two-stage transformation process. Fig. 11 depicts an overview of the document artifacts involved in the transformation process, as well as the flow relations between them. The leftmost part of the figure indicates how the original WS-BPEL process definition file and various XML fragment files serve as input for the *Template Generator* XSLT file. This Template Generator constitutes the first transformation step and turns the generic fragment templates into fragments tailored to the target process definition. The last transformation step injects the generated fragments into the original WS-BPEL process file.

### 6.4 Checking business activity constraints

The process transformation approach presented in Section 4 ensures runtime enforcement of Business Activity entailment constraints. For highly business- or security-critical systems we propose log analysis to additionally monitor that the process instances behave as expected (see, e.g., [40]). To check whether all constraints are fulfilled in the log data, we require an engine capable of querying the state of historical invocation data. As our framework is operating in a Web Services environment, XML is the prevalent data format and we focus mainly on XML tooling. We hence utilize XQuery [41] to continuously perform queries over the invocation logs stored in XML. To facilitate the handling of these queries, we use WS-Aggregation [42], a platform for event-based distributed aggregation of XML data.

Listing 4 prints exemplary log data that are emitted by the transformed business process and handled by WS-Aggregation. Each *log* element in the listing represents one invocation event.

Listing 3 prints the constraint enforcement queries, expressed as XQuery assertion statements that are expected to always yield a boolean *true* value. Lines 1–7 contain an excerpt of the constraint definitions in our scenario. For instance, the two tasks named *Get*_*Personal*_*Data* and *Assign*_*Physician* are in a role-binding relationship and hence combined in an element rbind. Moreover, the code binds the log elements from Listing 4 to the variable $logs (line 8). Finally, Listing 3 contains the four XQuery expressions used for enforcing constraints concerning SME tasks (lines 11–15), DME tasks (lines 17–19), subject-bindings (lines 22–25) and role-bindings (lines 27–30).

The four expressions use universal quantification (every… in… satisfies) to express assertions about pairs of tasks defined in the constraints list. The variables $*t1* and $*t2* refer to the names of the respective tasks. The query for SME loops over all pairs of SME tasks and ensures that the logs do not contain invocations for both tasks that use the same subject or the same role. The DME query tasks is similar, with the difference that only the subject is queried and additionally the *instanceID* attribute of the log entries is considered. Subject-binding is checked by ensuring that for all log entries of a particular process instance two tasks $*t1* and $*t2* are executed by the same subject. The role-binding query works analogously, but instead of using the *subject* attribute, here we require the *role* attribute to match for all *rbind*-connected tasks that occur in the same process instance.

## 7 Evaluation and discussion

In this section, we evaluate various aspects to highlight the benefits, strengths, and weaknesses of the presented solution. Five business processes with entailment constraints were selected to conduct the evaluation, including our example process from Section 2 and four additional processes from existing literature. The examples represent typical processes from different domains and cover all constraint types supported by our approach. The key properties of the evaluated processes are summarized in Table 3: *ID* identifies the process (*P*1 is our sample process), ∣*T*_*T*_∣ is the total number of task types per process, ∣*CT*_*T*_∣ is the number of task types associated with entailment constraints^5^, ∣*R*∣ is the number of roles defined in the scenario, ∣*S*∣ is the number of subjects used for the test, and ∣*HR*∣ is the number of senior-junior relationships in the role hierarchy^6^.

Although not all results of our evaluation are fully generalizable, they are arguably valid for a wide range of scenarios and SOA environments in general. An evident observation is that runtime enforcement of security constraints is computationally intensive, and therefore performance effects need to be taken into account. We also show that the proposed DSL greatly simplifies development of security-enabled WS-BPEL processes, which becomes apparent when comparing the number of code artifacts before and after automatic transformation. However, the approach also has certain limitations which we also want to document explicitly. Overall, our evaluation is organized in four parts: first, we evaluate the runtime performance in Section 7.1; second, in Section 7.2 we verify the behavior of secured processes when provided with valid and invalid authentication data^7^; third, Section 7.3 evaluates the WS-BPEL transformation procedure; fourth, in Section 7.4 we discuss current limitations in the framework and general threats to validity. The experiments in Sections 7.1, 7.2, 7.3 were executed on a machine with Quad Core 2.8 GHz CPU, 8 GB RAM, running Ubuntu Linux 9.10 (kernel 2.6.31–23).

### 7.1 Performance and scalability

For our scalability evaluation we have defined, deployed, and executed different process instantiations (based on the example in Section 2) in a Glassfish server (version 2.1.1) with WS-BPEL engine (version 2.6.0). Here, we are only interested in the net processing time of the Web service invocations, the duration of human tasks is not considered. Therefore, the execution of business operations (e.g., *Obtain X-ray Image* or *Decide On Treatment*) has zero processing time in our testbed.

**Fig. 12:**
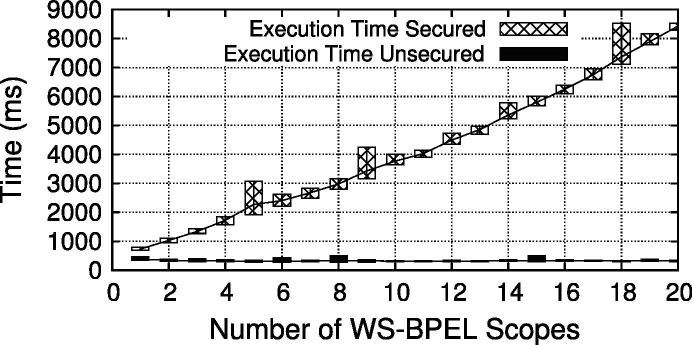
Process execution times – secured vs unsecured.

The WS-BPEL process has been deployed in different sizes (multiple scopes, one invoke task per scope), once with enforced security (i.e., annotated with security attributes, automatically transformed at deployment time), and once in an unsecured version. The deployed processes were executed 100 times and we have computed the average value to reduce the influence of external effects. Fig. 12 plots the execution time (minimum, maximum, average) for both the secured (top line) and the unsecured version (bottom line). The top/bottom of each box represents the maximum/minimum, respectively, and a trendline is drawn for the average value^8^. We observe that a single BusinessAction invocation in the unsecured version is very fast, whereas the secured version incurs a considerable overhead. The overhead is hardly surprising considering that for each business logic service the process needs to invoke the IdP and RBAC services, as well as apply and check XML signatures. However, the measured results indicate that the current implementation has potential room for additional optimization.

**Fig. 13:**
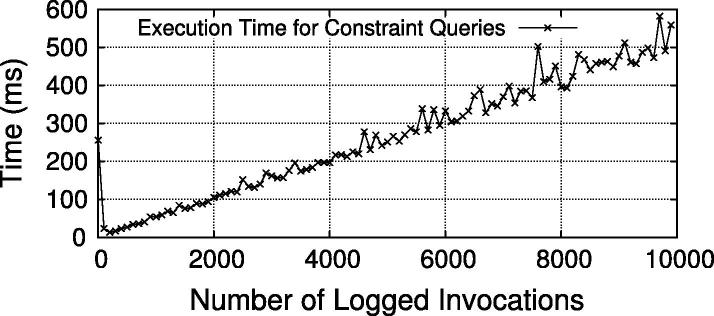
Execution time of constraint queries for increasing log data.

**Fig. 14:**
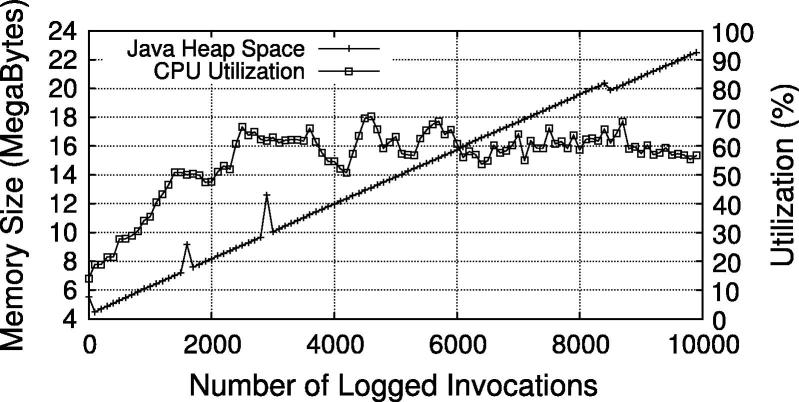
Resource consumption for constraint queries.

In addition to the end-to-end performance of the secured WS-BPEL process, we also evaluated the performance of enforcing the BusinessActivity constraints using the XQuery based querying approach. To that end, we stored 10,000 entries with SME, DME, SBind and RBind constraints to the invocation log and measured the time required to execute the four constraint queries in Listing 3. The results are illustrated in Fig. 13, which plots the time for every 100th invocation over time. As the testbed started cleanly from scratch, the first logged invocation(s) took longer (∼250 ms) because of internal initialization tasks in the log store and the WS-Aggregation query engine. Starting from the second data point (invocation 100), we see the query time increasing by around 6 ms per 100 queries. To provide an insight about resource consumption, the CPU utilization and Java heap space usage are plotted in Fig. 14. The slight fluctuations in heap space are due to Java’s garbage collection procedure. The four constraint queries are executed in parallel, but since they access a shared data structure with log data, internal thread synchronization is applied. Hence, CPU utilization reaches only a peak value of ∼70% (i.e., three of the four cores).

The increase of time is inherent to the problem of querying growing log data. We argue that query performance is feasible for medium-sized to even large scenarios. Firstly, as evidenced in Fig. 13, the execution time appears to grow only linearly (we have also performed a linear regression which showed almost perfect fit for *y* = 20 + 0.06*x*). The reason is that the queries are formulated in a way that always only the last added log entry needs to be compared to the other entries (hence, the queries are executed for each new log entry). Secondly, even for large logs (tens of thousands of entries) the execution time is still in a range of only a few seconds. If we extrapolate the test values for very huge logs (millions of entries), however, the current approach would take in the order of minutes, which may not be feasible for real-time processes. Hence, additional optimizations will be required for such very-large scale situations – a problem we actively tackle in our future work.

### 7.2 Reaction of the secured process to valid and invalid authentication data

In the second experiment, we utilize the five evaluation processes (see Section 7) to evaluate how our approach deals with authentication data of authorized and unauthorized users provided by the IdP service. As outlined in Section 4, the task of the IdP is solely to authenticate users, but the authorization in terms of RBAC constraints is enforced by the process instance (and, additionally, by the log data queries from Section 6.4). Hence, the reason for performing this experiment is to test the ability of the transformed business process to cope with unauthorized users who attempt to execute restricted process tasks. Moreover, we are interested in evaluating under which circumstances the RBAC rules may become overconstrained such that the process ends in a deadlock and is unable to continue. Our methodology in this experiment is to execute all possible instances of the test processes with respect to user authorization (given a set of subjects and process tasks, try each combination of subjects performing a task; see Section 7.2.1 for details). The chosen scenario processes have a feasible size to perform this full enumeration. We discuss detailed results based on the patient examination process (P1) in Section 7.2.2, and aggregated results over all five processes (P1-P5) in Section 7.2.3.

#### 7.2.1 Permutation of RBAC assignments

We define the domain [*T*_*T*_ → (*S* × *R*)] of RBAC assignment functions, where *T*_*T*_ is the set of BusinessAction task types, *S* is the set of subjects and *R* is the set of roles (cf. Section 3.1). The function defines which authentication data should be used for each task type. We then consider all possible permutations of function assignments in this domain, with the restriction that for each pair (*s*, *r*) ∈ *S* × *R* the subject *s* is directly associated with role *r*. To keep the domain small, inherited roles are not considered. For instance, in our scenario the pair (*Bob*,*Physician*) is included, but (*Bob*, *Staff*) is not considered, although *Bob* inherits the role *Staff* through *Physician*. Furthermore, note that SME constraints are checked at design-time when defining a process-related RBAC model. The static correctness rules ensure the consistency of the corresponding RBAC models at any time (see [14]). This means that it is not possible to define an inconsistent RBAC model where, for example, a subject or role possesses the right to execute two SME tasks. The respective RBAC model is then applied to make access decisions and to perform task allocations for all process instances. In other words, because for each process instance the allocation of the respective task instances is based on a consistent process-related RBAC model, it is not necessary to check the fulfillment of SME constraints again at runtime (see also [19]).

For each permutation one process instance has been executed, and the IdP service in the test environment is configured to return the authentication data that correspond to the respective permutation. The IdP keeps track of *getAuthenticationData* requests and registers how many duplicate requests are issued for any task type in each process instance. Recall that a duplicate request is always issued if the IdP provides authentication data of a non-authorized user. Thus, each duplicate *getAuthenticationData* request represents a blocked execution of a restricted task (which is the desired/expected behavior).

The purpose of this experiment setup is to empirically evaluate (1) whether the secured process correctly allows/denies access for valid/invalid provided credentials, respectively, and (2) how the platform deals with unresolvable conflicts (if the process deadlocks due to mutual exclusions). For instance, when *Get Personal Data* in our scenario has been invoked with (*Bob*, *Physician*) and the IdP provides (*John*, *Staff*) for *Assign Physician*, then it is required to get new authentication data because of a violated role-binding constraint. In this case, the IdP simply provides the next available authentication data, simulating the real-life situation that a new subject logs in after an unauthorized subject has been denied access. This procedure is repeated as long as new pairs of subject and role can be provided; if the process has unsuccessfully attempted to invoke a task with *all* possible combinations, the whole process terminates with a fault message. Note that this method of deadlock detection is suitable for our scenario which defines only a small number of subjects; for more advanced detection of deadlocks and unsatisfiable constraints we refer to related work [44], [45].

#### 7.2.2 Detailed discussion for the patient examination process

In our scenario, the domain (*S* × *R*) consists of the four pairs ((*John,* *Staff*), (*Jane,* *Physician*), (*Bob,* *Physician*), (*Alice,* *Patient*)), and six task types exist (∣*T*_*T*_∣ = 6). Hence, the total number of possible assignment function permutations is 4^6^ = 4096. However, the process structure allows to reduce this number because the decision node (whether the patient is in an emergency situation) splits the process into two possible execution paths (one path with five tasks and the other path with four tasks). The decision node has been simulated to uniformly use both of the two possible conditional branches. Therefore, in total only 4^5^ + 4^4^ = 1280 process instances have to be executed.

**Fig. 15:**
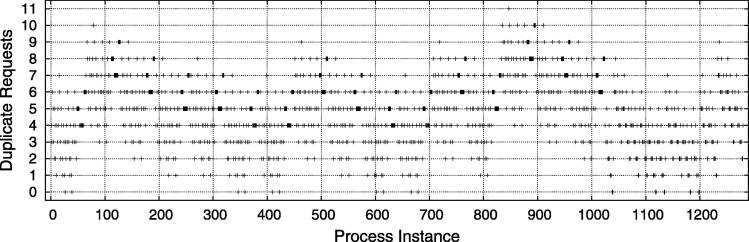
Blocked task executions per test process instance (patient examination scenario).

Fig. 15 illustrates the number of blocked authorization requests for each process instance. Considering the procedure of security enforcement (cf. Section 4), a blocked request means that the authentication data provided by the IdP violate any constraints (which is expected in many cases, since all permutations are tested). Table 4 summarizes the aggregated values: 20 of the 1280 generated RBAC assignments were completely valid from the start and no blocked requests were necessary. The remaining instances required between 1 and 11 blocked requests until a final state (successful or unsuccessful) is reached.

While there have been 1024 successful executions of the process, 256 failed instances had to be aborted because of deadlock situations. Deadlocks can result from the complex inter-dependencies of BusinessActivity access rules (see, e.g., [18], [46]). For instance, consider the operation sequence in Table 5. The deadlock is caused by the subject-binding between *Get Critical History* and *Decide On Treatment*, combined with the fact that both tasks can be executed by different roles (the former by *Patient* and *Physician*, and the latter only by *Patient*). In fact, all process executions in which the patient *Alice* executes *Get Critical History* lead to this conflicting situation. Note that the focus of this paper is to enforce RBAC constraints and to *detect* deadlocks^9^. In our future work we also investigate techniques to check the satisfiability of a certain process and *avoid* deadlocks in advance (see, e.g., [18], [44], [45], [47]).

**Fig. 16:**
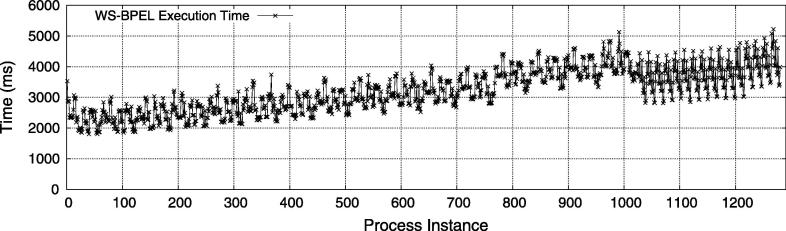
Execution time of secured BPEL process instances over time.

The same experiment setup has been used to measure the execution time of the secured process instances over time (Fig. 16). Again, we see a slight upwards trend in the processing time. The reasons for this trend are twofold. First, the more instances have executed, the more log data must be checked for constraint conflicts. Second, particularly for SME constraints an increasing number of log data increases the likelihood that the blocked requests need to be issued because the provided test authentication data are in a conflict with one or more previous invocations. The spikes in Fig. 16 indicate different execution times of instances with few versus many blocked requests (see also Fig. 15). Notice that the execution time shows a certain pattern between roughly 0 and 1000, and a different pattern between 1000 and 1280. These patterns are a direct result of the experiment design, because we first execute 1024 instances that follow the “emergency” path in the scenario process, and afterwards 256 instances that follow the “non-emergency” path.

#### 7.2.3 Aggregated results for all test processes

Table 6 summarizes the test results for the five test processes. The table contains the process *ID* that refers back to Table 3, the total number of executed instances which were generated from the RBAC assignment permutations, the number of deadlocks that occurred, the blocked requests (minimum/maximum/average) per process instance, and the aggregated execution time per instance. In general, the number of instances corresponds to $|S{|}^{\left|T_{T}\right|}$, except in cases where we can take advantage of the process structure to reduce the number of instances (i.e., 1280 instead of 4096 instances for P1). Process P4 has the highest number of instances (3125). The aggregated values are computed over all process instances; for example, the average number of blocked requests over all 1280 instances of process P1 is 4.8. The difference between minimum and maximum execution time depends on the executed tasks, and hence correlates strongly with the number of blocked requests. The maximum execution time was roughly 14 s (for an instance of process P4), and the shortest instance (of P1) executed within less than 2 s. Depending on the process definition and the chosen subjects, either all generated process instances were able to execute successfully (P3, P4, P5), or some instances deadlocked (P1, P2). Some process definitions are prone to deadlocking (e.g., 20% of P1’s possible instances lead to a deadlock), whereas in other processes deadlocks are not even possible. For instance, the tax refund process [16] (P4) was run with the smallest possible number of subjects (at least two clerks and three managers are required), but out of the 3125 instances (each subject tries to access each of the five task types, 5^5^ = 3125) not a single instance deadlocks. Even though satisfiability of access constraints at different points of the process execution can be determined algorithmically (see, e.g., [18]), we argue that it is equally important to test the running system, and to empirically verify the number of successful and blocked requests, as shown in this evaluation.

### 7.3 WS-BPEL transformation algorithm

Concerning the evaluation of the WS-BPEL transformation algorithm, we consider the same twenty test process definitions with different sizes described earlier in Section 7.1.

**Fig. 17:**
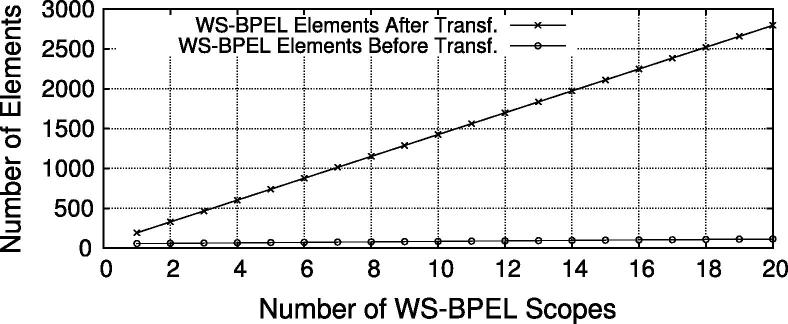
Different sizes of WS-BPEL processes before transformation (i.e., process annotated with RBAC DSL statements) and after transformation (i.e., process with injected security enforcement tasks).

Fig. 17 shows the number of WS-BPEL elements of the process definition before and after the automatic transformation. The results indicate that the size of the WS-BPEL definition rises with increasing number of scopes. While our test process with a single scope contains 33/115 WS-BPEL elements before/after transformation, the process definition for 10 scopes grows to 60/484 WS-BPEL elements before/after transformation, respectively. These numbers are determined by counting all XML (sub-) elements in the WS-BPEL file using the XPath expression count(//*). At the beginning of the transformation, 41 elements are added (import, partnerLink and variable declarations), and for each new scope 41 elements are added for the IAM task definitions (note that both values are 41 coincidentally). We observe that the ability to define security annotations in WS-BPEL keeps the process definition clear at design time. In fact, the additional code for security enforcement in WS-BPEL is often larger than the actual business logic. This can be seen as an indicator that our approach can reduce the development effort as compared to manual implementation, although we did not empirically evaluate this aspect in detail.

### 7.4 Limitations

In this section, we discuss the current limitations and weaknesses of our approach and the corresponding Web service technology projection. We also propose possible mechanisms and future work to mitigate the consequences and risks associated with these limitations.

• Parallel Process Flows: WS-BPEL provides the flow command for concurrent execution of tasks. Security enforcement of tasks that execute in parallel poses a challenge for various reasons. Firstly, if two tasks are started with mutually exclusive access rights, a race condition is created with respect to the first task to access the authentication token. Secondly, since we make use of “global” (process-instance-wide) variables, the injected IAM tasks for each single WS-BPEL invoke action are supposed to execute atomically and should not access these variables concurrently. To handle parallel execution, we hence propose to extend the injected IAM tasks with two additional tasks to acquire and release an exclusive lock when entering and leaving the critical region, respectively. Since BPEL does not provide a corresponding language construct, an external Web service is used to acquire the exclusive lock on a semaphore. For brevity and clarity, these additional synchronization tasks have not been added to the transformation in Section 4. In future work, we further plan to introduce more sophisticated synchronization using the WS-BPEL link mechanism.
• Deadlocking: If the RBAC policies are conflicting, the procedure for obtaining and checking user authentication data can end up in a deadlock that is unable to terminate with a successful result. To mitigate the effect of policy conflicts, it is therefore required to perform timely satisfiability checks. In Section 8 we discuss related work that focuses on this topic, in particular we refer to previous work in [18], [19], [46], [47].
• Single Point of Failure: Our Web service technology projection builds on the assumption that the IdP and Logging services operate reliably and continuously. An outage of any of these services would imply that the access control procedure cannot be performed in its entirety or that certain log data cannot be stored. Depending on the process definition at hand, the consequences can be more or less severe. The IdP service is the key component that provides the basis for user authentication. If it is unavailable, the secured execution fails. A possible strategy for certain application scenarios would be to define *break-the-glass* (BTG) rules (see, e.g., [48], [49], [50]) which allow to temporarily access the protected resources with fallback security settings, in order to provide for continuous operation. An outage of the Logging service is less severe, because it is strictly only required to perform a posteriori conformance checks of global constraints that may affect all (or at least multiple) process instances (see, e.g., [51]). Instance-specific constraints are local to a certain process instance and can be enforced by means of instance-specific log data stored in WS-BPEL variables (see Section 5).
• Security Token Hijacking: Malicious users may attempt to gain access to services they are not entitled to. Consider an attacker who intentionally does not follow the processing logic of the transformed process but invokes the target Web services directly. The attacker may obtain a SAML token by executing *getAuthenticationData*, which asserts its subject and role. Assume that the token is used in combination with the *instanceID* of an active process instance to invoke the *Decide On Treatment*; this situation must be avoided under any circumstances. To enforce the subject-binding with *Get Critical History* and other RBAC rules it is imperative that all access constraints are validated on the service side. In our architecture we hence require a policy enforcement point (PEP) which intercepts and analyzes all invocations.
• Invalid WS-BPEL Modification: For the approach to work reliably, it is important that the WS-BPEL definition should not be modified after the automated code transformation step. We therefore propose the use of a trusted deployment component which provides exclusive access to the business process execution engine. As part of transformation process the WS-BPEL file is signed with an XML signature [52], which is then checked by the deployment component to enforce integrity.
• Human Factors: In the end, a business process involving human labor can only be as safe and reliable as the persons who perform it. That is, control mechanisms such as mutual exclusion (e.g. to enforce the four-eyes principle) can provide a strong instrument for improving quality and reliability, but human errors can never be fully ruled out.

## 8 Related work

This section provides a discussion of related work in the area of model-driven IAM and their application to SOA business processes. Our analysis focuses on three main research areas: security modeling for Web service based systems, DSL-based security modeling, and techniques for incorporating runtime enforcement of security constraints into business processes.

### 8.1 Security modeling for web service based systems

Jensen and Feja [53] discuss security modeling of Web service based business processes, focusing on access control, confidentiality and integrity. Their approach is based on Event-driven Process Chains (EPCs) [54] and defines different security symbols that the process definitions are annotated with. Their implementation is integrated into the ARIS SOA Architect software, which is also able to transform the EPC model into an executable SOA business process. The paper describes the generation of WS-SecurityPolicy [55] policies, but does not discuss mutual exclusion and binding constraints in process-related RBAC models, nor does it discuss in detail how the process engine enforces the policies and constraints at runtime, which in contrast is a core part in our work.

Kulkarni et al. [56] describe an application of context-aware RBAC to pervasive computing systems. As the paper rightly states, model-level support for revocation of roles and permissions is required to deal with changing context information. The approach has a strong focus on dynamically changing context (e.g., conditions measured by sensors) and the associated permission (de-)activation. In our framework, context information is part of the RBAC model definitions (more details can be found in [21]). In this paper, the context information in the RBAC model has been abstracted from, but as part of our future work we plan to integrate the Business Activity model in [14] with context information (see also [57]).

Although our model does not directly build on the notion of trust, access control policies can also be established dynamically by deriving trust relationships among system participants [58]. Skoksrud et al. present Trust-Serv [59], a solution for model-driven trust negotiation in Web service environments. Similar to our approach, the policy enforcement is transparent to the involved Web services. Another similarity is that trust credentials (such as user identifier, address or credit card number) are exchanged iteratively throughout the process, which is also the case for the authentication credentials in our approach. However, trust-based policies in [59] are *monotonic* in the sense that additional trust credentials always add access rights and never remove existing ones, which is in contrast to access control in this paper, where the execution of tasks can activate entailment constraints which progressively narrow down the set of valid access control configurations.

Our approach was also influenced by Foster et al. [60] who present an integrated workbench for model-based engineering of service compositions. Their approach supports service and business process developers by applying formal semantics to service behavior and configuration descriptors, which can then be analyzed and checked by a verification and validation component. The policies enforced by the workbench are quite generally applicable and hence require developers to perform application specific modeling, whereas our proposed DSL and WS-BPEL annotations are tailored to the domain of RBAC and entailment constraints and arguably straight-forward to apply.

Seminal contributions in the context of modeling support for Web service based business processes are provided within the Web Services Modeling Framework (WSMF) by Fensel et al. [61], and the modeling ontologies that emerged from this project. For instance, security requirements can be modeled in WSMF by declaring the subject and role as input data and defining pre-conditions for all operations that require certain authentication data. In the previous years, the Semantic Web community has been pushing forward various ontologies to draw an ever more exact picture of the functionality exposed by Web services, in order to allow for sophisticated discovery, execution, composition and interoperation [62]. In fact, although not very frequently used in practice, semantically annotated Web services also allow for a more fine-grained definition of access control policies, from the interaction level down to the message level. Whereas annotations in semantic Web services are used mostly for reasoning purposes, the BPEL annotations used in our approach are utilized as metadata for runtime access control enforcement. Such business process model abstractions, which are the underpinning of semantic equivalence and structural difference, have been empirically studied in [63], and our approach can be seen as the reverse operation of abstraction (i.e., concretization) for the specific application domain of task-based entailment constraints.

Various other papers have been published that are related to our work or have influenced it, some of which are mentioned in the following. The platform-independent framework for Security Services named SECTISSIMO has been proposed by Memon et al. [64]. The conceptual novelty of this framework is the three-layered architecture which introduces an additional layer of abstraction between the models and the concrete implementation technologies. In contrast, our prototype only considers two layers (i.e. modeling of RBAC constraints and transformation of WS-BPEL code). However, the presented modeling concepts (see Section 3) as well as the model transformations (see Section 4) are independent from concrete implementation technologies too.

Lin et al. [65] propose a policy decomposition approach. The main idea is to decompose a global policy and distribute it to each collaborating party. This ensures autonomy and confidentiality of each party. Their work is particularly of relevance for cross-organizational definition of RBAC policies, as performed in our multi-hospital use case scenario. Currently, our prototypical implementation relies on a single, global RBAC Web service. However, we plan to adopt this complementary policy decomposition approach, which will allow each hospital to employ its own dedicated RBAC Web service.

### 8.2 DSL-based security modeling

An integrated approach for Model Driven Security, that promotes the use of Model Driven Architectures in the context of access control, is presented by Basin et al. [66]. The foundation is a generic schema that allows creation of DSLs for modeling of access control requirements. The domain expert then defines models of security requirements using these languages. With the help of generators these models are then transformed to access control infrastructures. However, compared to our approach, [66] does not address the definition of task-based entailment constraints.

The approach by Wolter et al. [36] is concerned with modeling and enforcing security goals in the context of SOA business processes. Similar to our approach, their work suggests that business process experts should collaboratively work on the security policies. They define platform independent models (PIM) which are mapped to platform specific models (PSM). At the PIM level, XACML and *AXIS 2*^10^ security configurations are generated. Whereas their approach attempts to cover diverse security goals including integrity, availability and audit, we focus on entailment constraints in service-based business processes.

A related access control framework for WS-BPEL is presented by Paci et al. in [67]. It introduces the *RBAC-WS-BPEL* model and the authorization constraint language *BPCL*. Similar to our approach, the BPEL activities are associated with required permissions (in particular, we associate permissions for invoke activities that try to call certain service operations). However, one main difference is related to the boundaries of the validity of user permissions: RBAC-WS-BPEL considers pairs of adjacent activities (*a*_1_ and *a*_2_, where *a*_1_ has a control flow link to *a*_2_) and defines rules among them, including separation of duty (*a*_1_ and *a*_2_ must execute under different roles) and binding of duty (*a*_1_ and *a*_2_ require the same role or user). As elaborated in previous work [21], our approach also allows to annotate scopes (groups of invoke tasks) in BPEL processes and hence to apply RBAC policies in a sequential, but also in a hierarchical manner.

XACML [68] is an XML-based standard to describe RBAC policies in a flexible and extensible way. Our DSL could be classified as a high-level abstraction that implements a subset of XACML’s feature set. Using a transformation of DSL code to XACML markup, it becomes possible to integrate our approach with the well-established XACML environment and tools for policy integration (e.g., [69]).

### 8.3 Runtime enforcement of security and other constraints in business processes

Various approaches have been proposed to incorporate extensions and cross-cutting concerns such as security features into business process models. Most notably, we can distinguish different variants of model transformation [70], [30] and approaches that use aspect-oriented programming [71].

A dynamic approach for enforcement of Web services Security is presented in [72] by Mourad et al. The novelty of the approach is mainly grounded by the use of Aspect-Oriented Programming (AOP) in this context, whereby security enforcement activities are specified as *aspects* that are dynamically woven into WS-BPEL processes at certain *join points*. Charfi and Mezini presented the AO4BPEL [73] framework, an aspect-oriented extension to BPEL that allows to attach cross-cutting concerns. The aspect-oriented language Aspects for Access Control (AAC) by Braga [74] is based on the same principle and is capable of transforming SecureUML [75] models into aspects. A main difference is that AAC does not operate on BPEL, but on Java programs, and can hence be applied directly to Java Web service implementations to enforce access control.

Essentially, our approach can be regarded as a variant of AOP: the weaved aspects are the injected IAM tasks, and join points are defined by security annotations in the process. A major advantage of our approach is the built-in support for SSO and cross-organizational IAM. An interesting extension could be to decouple security annotations from the WS-BPEL definition, to store them in a separate repository and to dynamically adapt to changes at runtime.

A plethora of work has been published on transformations and structural mappings of business process models. Most notably, our solution builds on work by Saquid and Orlowska [76], and Eder and Gruber [77] who presented a meta model for block structured workflow models that is capable of capturing atomic transformation actions. These transformation building blocks are important for more complex transformations, as in our case when multiple process fragments for enforcement of entailment constraints are combined for a single action in WS-BPEL. While this work focuses mainly on deployment time model transformations, other research also investigates runtime changes of service compositions. For instance, automatic process instrumentation and runtime transformation have previously been applied in the context of functional testing [78] and fault detection [79] for service-based business processes. Weber et al. [80] investigate security issues in adaptive process management systems and claim that such dynamicity increases the vulnerability to misuse. Our approach is adaptive in that it allows the “environment” (e.g., access policies) to change at runtime. However, we currently assume that the process definition itself does not change. In our ongoing research, we are complementing our approach with support for online structural process adaptation.

An important aspect of security enforcement is the way how constraint conflicts are handled at runtime. Consequently, our approach is related to a recent study on handling conflicts of binding and mutual exclusion constraints in business processes [46], [47]. Based on a formalization of process-related RBAC, this work proposes algorithms to detect conflicts in constraint definitions, as well as strategies to resolve the conflicts that have been detected. In our evaluation (see Section 7), we illustrated an example constraint conflict that lead to a deadlock and discussed how the platform is able to detect such conflicts. In order to anticipate and avoid deadlocks altogether, we will eventually integrate these algorithms with our RBAC DSL.

Although not necessarily concerned with security (i.e., access control) in the narrower sense, the area of Web service transaction processing [81], [82] and conversational service protocols [83], [84] is related to our work on secured business processes. Put simply, a transactional protocol is a sequence of operations with multiple participants that have a clearly defined role and need to collaboratively perform a certain task. Analogously, BusinessActivities are performed by subjects with clearly defined roles and limited permissions. One could argue that while the responsibility of transaction control is to ensure that all participants actually *do* perform their task, the main purpose of access control is to ensure that subjects *do not* perform tasks they are not authorized to. Amongst others, our approach was influenced by von Riegen et al. [82] who model distributed Web service transactions with particular focus on complex interactions where participants are restricted to only possess limited local views on the overall process. These limited views are comparable to our access control enforcement. Our approach also detects if a process instance is about to break the required conversational protocol (i.e., access control policies), in which case we apply a sequence of compensation actions [81] (e.g., repeat authentication or terminate instance due to deadlock).

## 9 Conclusion

We presented an integrated, model-driven approach for the enforcement of access control policies and task-based entailment constraints in distributed service-based business processes. The approach is centered around the DSL-driven development of RBAC policies and the runtime enforcement of the resulting policies and constraints in Web services based business processes. Our work fosters cross-organizational authentication and authorization in service-based systems, and facilitates the systematic development of secured business processes. From the modeling perspective, the solution builds on the BusinessActivity extension – a native UML extension for defining entailment constraints in activity diagrams. We provided a detailed description of the procedure to transform design-time BusinessActivity models into standard activity models that enforce the access constraints at runtime. Based on a generic transformation procedure, we discussed our implementation which is based on WS-BPEL and the Web services framework.

Our approach based on BusinessActivities allows to abstract from the technical implementation of security enforcement in the design time view of process models. The detailed evaluation of the process transformation has shown that process definitions with injected tasks for security enforcement grow considerably large. In fact, the additional code for security enforcement in WS-BPEL is often larger than the actual business logic. This can be seen as an indicator that our approach can reduce the development effort as compared to manual implementation, although we did not empirically evaluate this aspect in detail.

Our extensive performance evaluation has illustrated that the proposed runtime enforcement procedures operate with a slight overhead that scales well up to the order of several ten thousand logged invocations. We can conclude that the overhead consists of three main parts: (1) the approach builds on digital signatures for ensuring message integrity, (2) the process determines the role and permissions of the currently executing user, which results in additional requests and increased execution time, and (3) the enforcement of entailment constraints requires querying the log traces of previous executions of the process. Note that the overhead for (1) and (2) does not increase over time (with rising number of process executions), whereas the overhead for (3) inherently rises because the log traces are accumulating over time, and more data have to be evaluated.

The implementation of our prototype still has limitations, and we discussed strategies to improve some of these limitations in future work. For instance, advanced synchronization mechanisms are required for business processes with highly parallel processing logic. Moreover, the query mechanism that checks security constraints for validity needs to be further optimized for very large log data sets (in the order of millions of invocations). We envision advanced data storage and compression techniques, as well as optimized query mechanisms to further reduce this increase of overhead over time. In our ongoing work we also investigate the use of additional security annotations and an extended view of context information. Finally, we plan to shift from a process-centric to a more data-centric view and integrate the concept of entailment constraints to our recent work on reliability in event-based data processing [85] and collaborative Web applications [86].

## Figures and Tables

**Listing 5:**
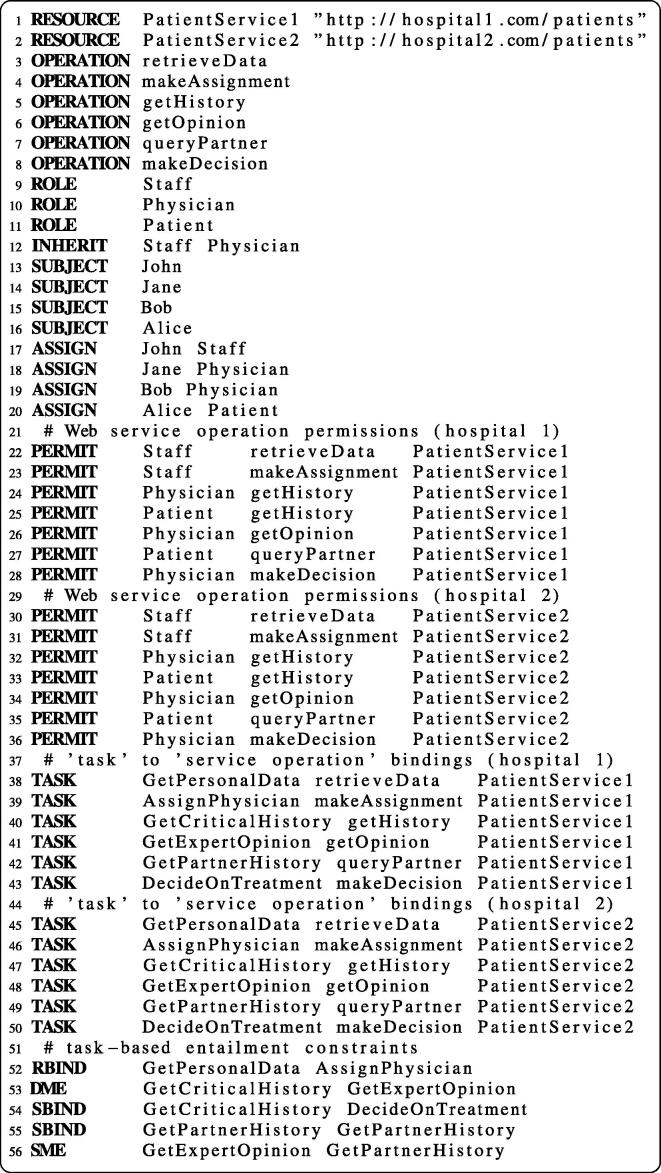
Exemplary RBAC DSL statements for hospital scenario.

**Listing 1:**
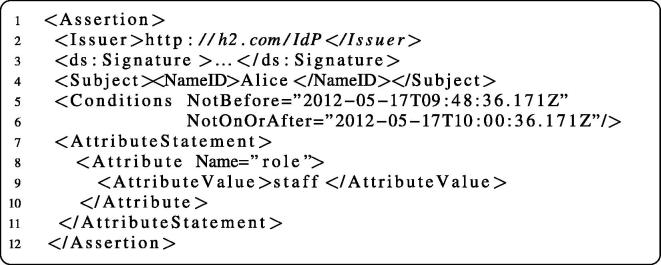
Exemplary SAML assertion carrying subject and role information.

**Listing 2:**
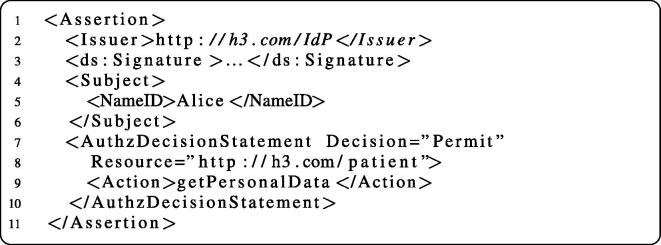
Exemplary SAML authorization decision.

**Listing 3:**
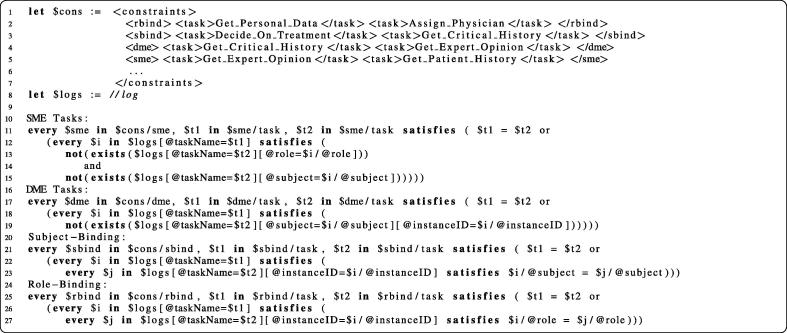
XQuery assertion expressions for enforcing business activity constraints.

**Listing 4:**
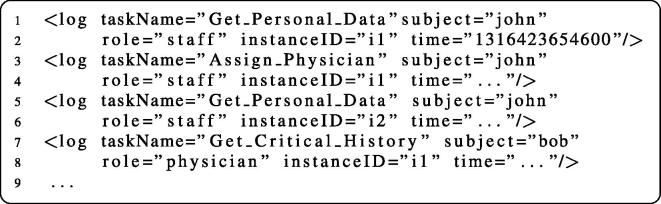
Format of invocation data logged as events.

**Table 1:** Semantics of RBAC DSL statements.

RBAC DSL statement	Effect
**RESOURCE***name* [*description*]	Define new resource
**OPERATION***name* [*description*]	Define new operation
**SUBJECT***name* [*description*]	Define new subject
**ROLE***name* [*description*]	Define new role
**ASSIGN***subject role*	Assign role to subject
**INHERIT***juniorRole seniorRole*	Let senior role inherit a junior role
**PERMIT***role operation resource*	Allow a role to execute a certain operation on a specific resource

**TASK***name operation resource*	Define operation-to-task mapping
**DME***task*1 *task*2	Define dynamic mutual exclusion (DME)
**SME***task*1 *task*2	Define static mutual exclusion (SME)
**RBIND***task*1 *task*2	Define role-binding (RBind)
**SBIND***task*1 *task*2	Define subject-binding (SBind)

**Table 2:** Mapping of RBAC DSL statements to WS-BPEL DSL statements.

DSL statement	WS-BPEL DSL statement
**DME***task*1 *task*2	<invoke name = “*task*1” **rbac:dme** = “*task*2” ../ >
**SME***task*1 *task*2	<invoke name = “*task*1” **rbac:sme** = “*task*2” ../ >
**SBIND***task*1 *task*2	<invoke name = “*task*1” **rbac:sbind** = “*task*2” ../ >
**RBIND***task*1 *task*2	<invoke name = “*task*1” **rbac:rbind** = “*task*2” ../ >

**Table 3:** Characteristics of business processes used in the evaluation.

ID	Name	∣T_T_∣	∣CT_T_∣	∣R∣	∣S∣	∣HR∣
P1	Patient examination	7	6	3	4	1
P2	Purchase order [43]	6	4	2	3	1
P3	Paper review [14]	5	4	3	5	0
P4	Tax refund [16]	5	4	2	5	0
P5	Credit application [14]	5	3	2	4	1

**Table 4:** Process executions with permutations of *T*_*T*_ → (*S* × *R*) assignments.

Result outcome	Instances
No blocked requests	20
1 Blocked request	56
2 Blocked requests	108
3 Blocked requests	163
4 Blocked requests	228
5 Blocked requests	232
6 Blocked requests	210
7 Blocked requests	140
8 Blocked requests	80
9 Blocked requests	32
10 Blocked requests	10
11 Blocked requests	1

Successful execution	1024
Failed (Deadlocked)	256

Total instances	1280

**Table 5:** Operation sequence leading to a constraint conflict (Deadlock).

Task	Sub.	Role	Effect
*Get Personal Data*	*John*	*Staff*	Role *Staff* must *Assign Physician*
			*John* must *Assign Physician*
*Assign Physician*	*John*	*Staff*	–
*Obtain X-ray Image*	*Bob*	*Physician*	–
*Get Critical History*	*Alice*	*Patient*	*Alice* must not *Get Expert Opinion*
			*Alice* must *Decide On Treatment*
*Get Expert Opinion*	*Jane*	*Physician*	–
*Decide On Treatment*	*?*	*?*	Deadlock, because the bound subject *Alice* is not permitted

**Table 6:** Aggregated test execution results of the five evaluated processes.

ID	Instances	Deadlocks	Blocked requests			Execution time (ms)		
			Min.	Avg.	Max.	Min.	Avg.	Max.
P1	1280	256	0.0	4.8	11.0	1802.0	3199.6	5222.0
P2	729	243	0.0	3.3	7.0	3990.0	5009.0	8881.0
P3	625	0	0.0	3.6	8.0	3444.0	5464.8	8057.0
P4	3125	0	0.0	6.9	16.0	2984.0	8356.6	14363.0
P5	64	0	0.0	1.8	4.0	2799.0	3070.1	5530.0

## Appendix A RBAC DSL statements for scenario process

Listing 5 contains the complete access control configuration of the Patient Examination scenario process (two involved hospitals), expressed using RBAC DSL statements.

## Footnotes

1 http://www.infosys.tuwien.ac.at/prototype/SeCoS/.

2 https://glassfish.dev.java.net/.

3 http://santuario.apache.org/.

4 http://commons.apache.org/bsf/.

5 *CT*_*T*_ = {*t* ∈ *T*_*T*_∣*sb*(*t*) ≠ ∅  ∨ *rb*(*t*) ≠ ∅ ∨ *sme*(*t*) ≠ ∅ ∨ *dme*(*t*) ≠  ∅}

6 *HR* = {(*s*, *j*) ∈ *R* × *R*∣*j* ∈ *rh*(*s*)}

7 Note that all processes from Table 3 where implemented and evaluated with the same rigor. However, we do believe that certain parts of our evaluation are best explained in detail based on a single process. Therefore, Sections 7.1, 7.2 exemplarily discuss the results from the patient examination example. This discussion applies analogously to the other processes from Table 3. The aggregated results for all processes are discussed in Section 7.2.3.

8 The standard deviation was in the range of 39.21 to 413.69 ms (lowest and highest values are for processes with 1 scope and 18 scopes, respectively) for the secured version, and in the range of 10.38 to 58.78 ms (for 13 scopes and eight scopes, respectively) for the unsecured version.

9 Note that the deadlocks in our evaluation result from the fact that we automatically generate and execute all possible process instances (see Section 7.2). Because our process-related RBAC models adhere to the static and dynamic consistency requirements defined in [14], [19] the resulting RBAC models are always consistent. However, even though we always have consistent models, it is still possible that a certain process is not satisfiable (see, e.g., [44], [45]).

10 http://axis.apache.org/axis2/java/core/.
